# Tofacitinib versus Biologic Treatments in Moderate-to-Severe Rheumatoid Arthritis Patients Who Have Had an Inadequate Response to Nonbiologic DMARDs: Systematic Literature Review and Network Meta-Analysis

**DOI:** 10.1155/2017/8417249

**Published:** 2017-03-09

**Authors:** Evelien Bergrath, Robert A. Gerber, David Gruben, Tatjana Lukic, Charles Makin, Gene Wallenstein

**Affiliations:** ^1^Mapi, Boston, MA, USA; ^2^Pfizer Inc., Groton, CT, USA; ^3^Pfizer Inc., New York City, NY, USA

## Abstract

*Objective*. To compare the efficacy and tolerability of tofacitinib, an oral Janus kinase inhibitor for the treatment of rheumatoid arthritis (RA), as monotherapy and combined with disease-modifying antirheumatic drugs (DMARDs) versus biological DMARDs (bDMARDs) and other novel DMARDs for second-line moderate-to-severe rheumatoid arthritis (RA) patients by means of a systematic literature review (SLR) and network meta-analysis (NMA).* Methods*. MEDLINE®, EMBASE®, and Cochrane Central Register of Controlled Trials were searched to identify randomized clinical trials (RCTs) published between 1990 and March 2015. Efficacy data based on American College of Rheumatology (ACR) response criteria, improvements in the Health Assessment Questionnaire Disability Index (HAQ-DI) at 6 months, and discontinuation rates due to adverse events were analyzed by means of Bayesian NMAs.* Results*. 45 RCTs were identified, the majority of which demonstrated a low risk of bias. Tofacitinib 5 mg twice daily (BID) and 10 mg BID monotherapy exhibited comparable efficacy and discontinuation rates due to adverse events versus other monotherapies. Tofacitinib 5 mg BID and 10 mg BID + DMARDs or methotrexate (MTX) were mostly comparable to other combination therapies in terms of efficacy and discontinuation due to adverse events.* Conclusion*. In most cases, tofacitinib had similar efficacy and discontinuation rates due to adverse events compared to biologic DMARDs.

## 1. Introduction

RA is a chronic autoimmune and inflammatory disease with no cure which leads to inflammation of the joints and surrounding tissues. It affects approximately 0.5–1% of the adult population worldwide [1]. RA afflicts nearly 2 million adults in the United States [2].

The primary goal of RA therapy is to maximize patients' long-term health-related quality of life through the control of symptoms, prevention of structural damage, and normalization of function [3, 4]. Current pharmacological therapies include nonbiologic and biologic DMARDs. Initial treatment consists of traditional, nonbiologic DMARDs, such as MTX or sulfasalazine (SSZ).

Patients who are intolerant or experience moderate/high disease activity despite traditional, nonbiologic DMARDs (inadequate response [DMARD-IR]) are often treated with a biologic DMARD as a second-line treatment. Biologic DMARDs include tumor necrosis factor (TNF) inhibitors, selective T-cell costimulatory modulators, or interleukin-6 (IL-6) receptor antagonists or the IL-1 receptor antagonists [5, 6]. Biologic therapies can be added to existing conventional DMARD therapy or be used as monotherapy. The majority of the data support use of biologic DMARDs in combination with csDMARDs over monotherapy; however, monotherapy has also been shown to be efficacious for some biologics [7–9]. All biologic DMARDs are administered intravenously or subcutaneously.

Tofacitinib is an oral Janus kinase inhibitor for the treatment of rheumatoid arthritis. Tofacitinib is included in the 2015 ACR Guideline for the treatment of RA, which recommends tofacitinib as a second-line option (after disease-modifying antirheumatic drug [DMARDs]) as either monotherapy or combination therapy [10]. JAKs are a subgroup of the nonreceptor protein tyrosine kinases required for type I and type II cytokine receptor signaling, which play a key role in immune cell development, activation, and homeostasis [11, 12]. By inhibiting JAKs, tofacitinib may modulate leukocyte recruitment, activation, and effector cell function at sites of inflammation. In recent randomized clinical trials (RCTs), tofacitinib 5 mg and 10 mg BID as monotherapy or in combination with MTX or other nonbiologic DMARDs demonstrated clinical improvements over placebo or MTX in adult patients with moderate-to-severe RA [13–23].

Evidence-based treatment decisions require comparisons of available therapies. The currently available evidence base for efficacy of biologic DMARDs in nonbiologic DMARD-IR patients consists of many RCTs. However, none of the studies include all interventions simultaneously. Most trials are placebo-controlled and very few head-to-head comparisons of two biologic DMARDs are available.

An NMA, or multiple-treatment meta-analysis, can synthesize a network of RCTs and allow inferences to be made for comparisons not previously studied directly [23–26]. Even when direct evidence is available, combining this with indirect comparisons in an NMA may yield more refined and precise estimates for relative treatment effects [25, 26].

The objective of the current study was to compare tofacitinib 5 mg and 10 mg BID either as monotherapy or combined with DMARDs relative to bDMARDs currently approved or being considered for approval for second-line moderate-to-severe RA patients by means of a SLR and NMA of RCT evidence.

Outcomes of interest were ACR20/50/70 response criteria and change in HAQ-DI score at 24 weeks and treatment discontinuation due to adverse events.

## 2. Materials and Methods

### 2.1. Identification and Selection of Studies

A SLR was performed to identify RCTs evaluating the efficacy and discontinuation rates due to adverse events of biologic DMARDs either as monotherapy or in combination with a nonbiologic DMARD in DMARD-IR RA patients, published between 1990 and March 2015. MEDLINE, EMBASE, and Cochrane Central Register of Controlled Trials (CENTRAL) databases were searched simultaneously using Ovid® for articles published in English. Search terms included a combination of free-text and Medical Subject Heading (MESH) terms relevant to RA, biologic treatment, and RCTs (see Appendix: A. Search strategy). In parallel to the SLR, a review of conference proceedings from ACR and EULAR congresses in 2013 and 2014 was conducted.

Two reviewers independently evaluated each identified study against the following predetermined selection Population, Intervention, Comparator, Outcomes, Study Design (PICOS) criteria. Discrepancies were resolved by discussion and, if needed, by involvement of a third researcher.

#### 2.1.1. Population

*DMARD-IR Patients*. Trials were excluded if patients were required to fail at least two or more nonbiological DMARDs or if patients had received nonbiological DMARDs with no indication of an inadequate response or failure.

#### 2.1.2. Interventions

They included treatments approved and novel therapies of interest currently in development and seeking approval, including abatacept, adalimumab, anakinra, certolizumab pegol, etanercept, golimumab, infliximab, tocilizumab, baricitinib (investigational), and tofacitinib, alone or in combination with MTX, or other nonbiologic DMARDs.

#### 2.1.3. Comparisons

*Placebo or One of the Aforementioned Regimens*. Comparisons were excluded if only different dosages of the same intervention were given or if patients were receiving a single specified intervention but with a variety of concomitant background treatments.

#### 2.1.4. Outcomes/Endpoints

They included ACR response criteria, HAQ-DI, and rates of discontinuation due to adverse events [27, 28].

#### 2.1.5. Study Design

 The study design is RCTs (phase II and above).

### 2.2. Data Extraction

For each study meeting the selection criteria, details were extracted on study design, study population characteristics, interventions, and the following outcomes: the number of patients with at least 20%, 50%, or 70% improvement in ACR criteria (ACR20, ACR50, or ACR70, resp.), change from baseline in HAQ-DI, all assessed at 24 weeks' follow-up, and discontinuation from the trial due to adverse events throughout the study. The validity of each trial identified by the SLR was assessed using the “quality assessment of the study according to the Centre for Reviews and Dissemination of the University of York” [29] (see Appendix: B. Quality Assessment Checklist and Results). ACR criteria require a predefined improvement (at least 20%, 50%, or 70%) in both tender (TJC) and swollen (SJC) joint counts and in at least three of the following parameters: physician global assessment of disease, patient global assessment of disease, patient assessment of pain, C-reactive protein (CRP) (or erythrocyte sedimentation rate [ESR]), and degree of disability according to HAQ-DI [27]. The HAQ-DI assesses the level of an individual's functional ability and includes questions related to fine movements of the upper extremity, locomotor activities of the lower extremity, and activities involving both upper and lower extremities. The scale ranges from zero (without any difficulty) to three (unable to do) [28]. Global assessments were measured on a visual analog or Likert scale. Discontinuation was analyzed by means of a cloglog model with a binomial likelihood.

### 2.3. Analysis

Efficacy and discontinuation due to adverse events data were combined across studies by means of an NMA [23–26]. Agents used as monotherapy, or in combination with MTX or DMARDs only, were considered different treatments in one NMA. Therefore, no assumption was made regarding a constant additive effect of MTX across agents, allowing comparison of monotherapy and combination therapy. The primary outputs were pooled relative effect estimates of (a) each agent as monotherapy versus placebo, (b) each agent in combination with MTX versus placebo in combination with MTX, and (c) each agent in combination with any background DMARD versus placebo in combination with any background DMARD.

Bayesian NMA models were used to analyze the created data set for the outcomes of interest in order to simultaneously synthesize the results of the included studies and to obtain treatment effects [23, 26, 30, 31]. NMAs within the Bayesian framework involve data, a likelihood distribution, a model with parameters, and prior distributions [32, 33]. The model relates the data from the individual studies to basic parameters reflecting the (pooled) relative treatment effect of each intervention compared to an overall reference treatment, that is, placebo. Based on these basic parameters, the relative efficacy between each of the competing interventions was obtained.

For binary outcomes (ACR response and discontinuation due to adverse events), a logistic regression model with a binomial likelihood distribution was used. For continuous outcomes (change from baseline [CFB] in HAQ-DI), linear models with normal likelihood distributions were used. An NMA relies on the assumption that there are no differences in the distribution of modifiers of the relative treatment effects across comparisons. For each outcome, fixed- and random-effects models were compared in terms of the goodness of fit to the data and calculated as the posterior mean residual deviance. The deviance information criterion (DIC) provides a measure of model fit that penalizes model complexity [34]. The random-effects model resulted in the lowest DIC and was considered appropriate for the synthesis of available evidence.

The results of the NMA provide relative treatment effects for each treatment versus placebo. In order to transform the odds ratio (OR) for ACR into an expected response rate, the ORs of each regimen relative to placebo were combined with the average estimate of the odds of response with placebo across studies.

To avoid influence of prior distributions required for Bayesian analyses on the outcomes, noninformative prior distributions (vague priors) were used. Prior distributions of the relative treatment effects (i.e., log OR of ACR response and log rate ratio for discontinuation) and difference between treatments for HAQ-DI CFB were normal with mean of 0 and variance of 10,000. Where the evidence network had too few studies and thus scarce support, we specified fewer vague priors. OpenBUGS statistical software [35] was used for the analyses [36]. Summary statistics are presented for the relative treatment effects of each intervention. In addition to point estimates reflecting the most likely value, 95% credible intervals (95% CIs) reflecting the range of true underlying effects with 95% probability are presented.

## 3. Results and Discussion

### 3.1. Results

#### 3.1.1. Study Identification

**Figure 1:**
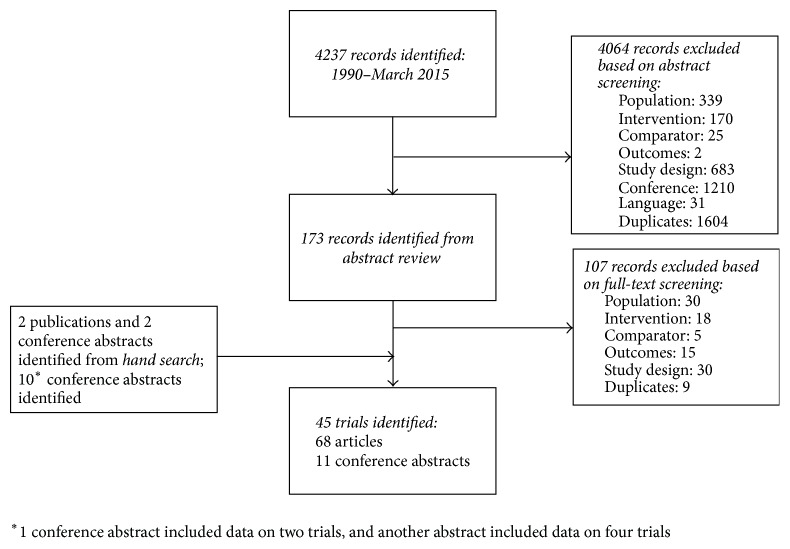
Flow diagram.

The literature search generated 4,237 citations from three databases (Figure 1). The first review of abstracts excluded 4,064 citations, primarily because the populations studied and/or study design did not meet the selection criteria. Review of the remaining 173 full-text reports excluded 107 publications, primarily because populations and/or study design did not meet the selection criteria. In parallel with the SLR, an abstract search of the two most recent ACR and European League Against Rheumatism (EULAR) congresses (2013 and 2014) was also conducted, and 10 conference abstracts were included. Further, two publications and two conference abstracts identified via hand search were included. In all, 68 full-text articles and 11 conference abstracts corresponding to 45 RCTs were identified (Figure 1). Top-line results of this SLR and NMA have been published previously [37].

#### 3.1.2. Evidence Base

A carefully conducted feasibility assessment was performed in order to ensure that the included studies were broadly comparable in terms of study design, patient characteristics, and treatment characteristics across the trials. The included evidence base was deemed broadly comparable, since no significant imbalances in relative treatment effect modifiers across comparisons were observed.

**Figure 2:**
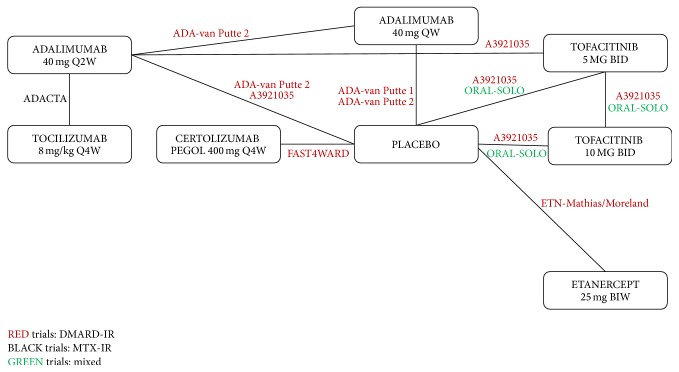
Monotherapy evidence network.

Figure 2 presents the corresponding monotherapy network, the combination therapy network, and the MTX combination therapy network, respectively, of all studies. Most studies were double-blind, parallel RCTs. The majority of trials were multicentered and multinational: most studies included patient populations predominantly from Europe and North America, although some studies also included patients from South America and Asia.

The majority of studies adopted similar eligibility criteria: adult patients with the diagnosis of RA based on ACR 1987 revised classification criteria, with active disease despite previous treatment with nonbiologic DMARDs, including MTX. Active-disease definitions varied around the minimum number of required TJC and SJC (6–12 of each) and also around the minimum levels of ESR (10 mm/h and 28 mm/h) and CRP (1–7 mg/dL). Some also required patients to report morning stiffness of ≥45-minute duration. Not all studies reported whether RA disease duration and DMARD treatment duration determined eligibility.

In RCTs evaluating efficacy of biologics in combination with a nonbiologic DMARD, MTX was the background treatment of choice. Of the 45 trials identified, 11 second-line trials required patients to be DMARD-IR, while 28 trials required patients to be MTX-IR. Six trials included patients who experienced an inadequate response to traditional DMARDs or biologic DMARDs (bDMARD-IR). Of the 45 included trials, only 18 restricted study inclusion to include second-line patients who had not received a third-line therapy. In addition, 15 trials did not report any information on patient's prior third-line treatment experiences or explicitly exclude patients who had previously taken third-line therapies.

Supplementary Table  1 (in Supplementary Material available online at https://doi.org/10.1155/2017/8417249) summarizes patient characteristics in the identified RCTs. Mean age ranged from 47 to 58.9 years. Patients were predominantly female (range: 43.3%–91.1%) and white (range: 44.4%–100%). Disease duration ranged from 0.7 to 15 years. ESR ranged from 24.38 to 60 mm/h; SJC ranged from 8.5 to 25; and TJC ranged from 12.9 to 35.5.

Most of the studies demonstrated a low risk of bias as assessed using the “quality assessment of the study according to the Centre for Reviews and Dissemination of the University of York” [29].

**Figure 3:**
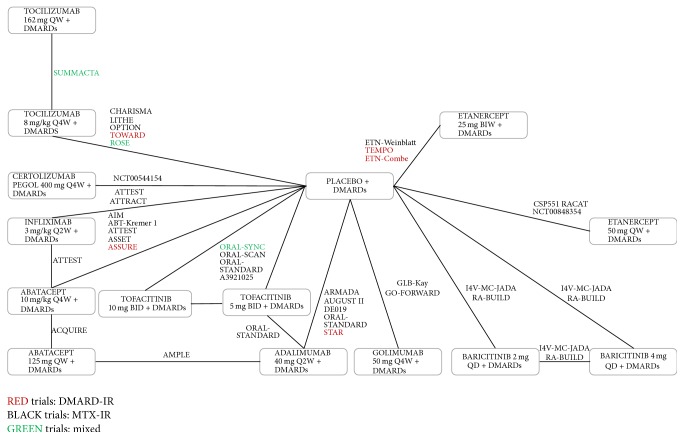
Combination therapy evidence network.

**Figure 4:**
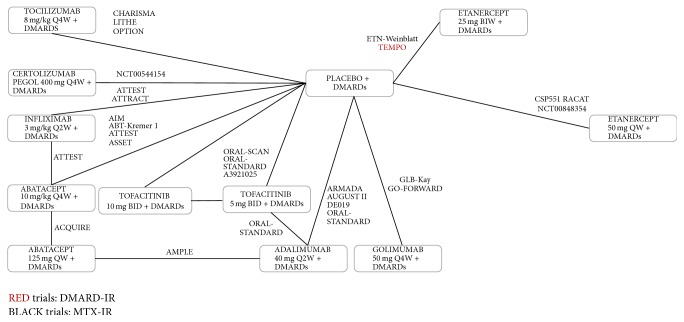
MTX combination therapy evidence network.

Overall, the networks are good reflection for all endpoints studied at 24 weeks (Figures 2–4). A link between two treatments in the network reflects direct evidence (i.e., ≥1 RCT) for that pairwise comparison. A path between two interventions consisting of ≥2 links reflects an indirect estimate for that contrast.

#### 3.1.3. Monotherapy

*Signs and Symptoms: ACR20/50/70*. Results of the NMAs for agents as monotherapy relative to tofacitinib are presented in Supplementary Figures 1−3. Tofacitinib 5 mg was comparable to the other monotherapies at 24 weeks in terms of ACR20 and ACR70 response rates, while tofacitinib 10 mg had more effective ACR20/50/70 response rates compared to placebo and was comparable to other monotherapies. Tofacitinib 5 mg demonstrated both greater efficacy than placebo and comparability to other monotherapies for ACR50 responses (see Table 1).

*Physical Functioning, as Measured by the HAQ-DI*. NMA of the CFB in HAQ-DI at 24 weeks including monotherapies was not feasible due to a lack of data.

*Discontinuation due to Adverse Events*. Tofacitinib 5 mg BID-related withdrawals due to adverse events were favorable to twice weekly adalimumab 40 mg (Q2W), comparable to the other monotherapies, and less likely to occur compared to placebo, certolizumab 400 mg every 4 weeks (Q4W), tocilizumab 8 mg/kg Q4W, and tofacitinib 10 mg BID. Withdrawals due to adverse events with tofacitinib 10 mg BID were comparable to all monotherapies. Furthermore, withdrawals due to adverse events were less likely to occur with tofacitinib 10 mg BID compared to adalimumab 40 mg Q2W, adalimumab 40 mg once weekly (QW), and tocilizumab 8 mg/kg Q4W (see Supplementary Figure 4).

#### 3.1.4. Combination Therapy

*Signs and Symptoms: ACR20/50/70*. Results of combination therapy with DMARDS relative to tofacitinib with DMARDs based on random-effects NMA are summarized in Table 2. For all ACR20/50/70 responses at 24 weeks, both tofacitinib 5 and 10 mg BID + DMARDs were more effective than placebo + DMARDs and showed comparable responses to other combination therapies (Table 2). Additionally, for the ACR70 responses, tofacitinib 5 mg BID + DMARDs and 10 mg BID + DMARDs were both more effective than certolizumab 400 mg Q4W + DMARDs. The ACR20 response likely favored tofacitinib 10 mg BID + DMARDs over etanercept 50 mg QW + DMARDs, abatacept 10 mg/kg Q4W + DMARDs, and infliximab 3 mg/kg Q8W + DMARDs (see Supplementary Figure  5–7).

Likewise, the ACR50 response at 24 weeks indicated likely favorability for both tofacitinib 5 mg + DMARDs and tofacitinib 10 mg BID + DMARDs over baricitinib 2 mg QD + DMARDs. Tofacitinib 10 mg BID + DMARDs was also likely favorable compared to etanercept 50 mg QW + DMARDs, abatacept 125 mg QW + DMARDs, and infliximab 3 mg/kg Q8W + DMARDs (See Table 2).

For the ACR70 response at 24 weeks, random-effects NMA comparison data showed adalimumab 40 mg Q2W + DMARDs and abatacept 10 mg/kg Q4W + DMARDs combination treatments to be likely less favorable than both tofacitinib 5 mg BID + DMARDs and tofacitinib 10 mg BID + DMARDs. Additionally, tofacitinib 10 mg BID + DMARDs showed likely favorability over etanercept 25 mg + DMARDs BID, etanercept 50 mg QW + DMARDs, infliximab 3 mg/kg Q8W + DMARDs, baricitinib 2 mg QD + DMARDs, and tofacitinib 5 mg + DMARDs (see Table 2).

*Physical Functioning: HAQ-DI*. The modelled change from baseline in HAQ-DI was greatest for tofacitinib 10 mg BID + DMARDs (see Supplementary Figure 8).

*Discontinuation due to Adverse Events*. Tofacitinib 5 mg BID + DMARDs and tofacitinib 10 mg + DMARDs were less favorable than placebo + DMARDs and abatacept 125 mg QW + DMARDs, but they were comparable to the other combination therapies with respect to withdrawals due to adverse events (see Supplementary Figure 9).

#### 3.1.5. MTX Combination Therapy

*Signs and Symptoms: ACR20/50/70*.  Table 3 outlines results of the NMAs for agents in combination with MTX relative to tofacitinib plus MTX. Both tofacitinib 5 mg BID + MTX and tofacitinib 10 mg BID + MTX showed a more effective response than placebo + MTX and were comparable to all other MTX combination therapies in terms of ACR20 and ACR50 at 24 weeks. Furthermore, odds ratios for tofacitinib 10 mg BID + MTX showed likely favorability over etanercept 50 mg QW + MTX, abatacept 10 mg/kg Q4W + MTX, and infliximab 3 mg/kg Q8W + MTX for ACR20/50 at 24 weeks (see Table 3).

In terms of ACR70 response at 24 weeks, both tofacitinib 5 mg BID + MTX and tofacitinib 10 mg BID + MTX were more effective than placebo + MTX and certolizumab 400 mg Q4W + MTX. Additionally, they were comparable to all other MTX combination therapies. Both tofacitinib treatment dosages plus MTX were likely to be favorable over adalimumab 40 mg Q2W + MTX, etanercept 25 mg + MTX BID, and abatacept 10 mg/kg Q4W + MTX. Tofacitinib 10 mg BID + MTX was also likely more favorable over additional MTX combination treatments etanercept 50 mg QW + MTX, abatacept 125 mg QW + MTX, infliximab 3 mg/kg Q8W + MTX, and tofacitinib 5 mg BID + MTX (see Supplementary Figure  10–12).

For physical functioning as measured by the HAQ-DI, tofacitinib 10 mg BID + MTX showed the greatest improvement in HAQ-DI (see Supplementary Figure 13).

Regarding discontinuation due to adverse events, both tofacitinib BID dosages in combination with MTX were likely to be less favorable than placebo + MTX, etanercept 25 mg BIW + MTX, abatacept 125 mg QW + MTX, and golimumab 50 mg Q4W + MTX. Tofacitinib 5 mg BID + MTX was also likely to be less favorable than etanercept 50 mg QW + MTX and abatacept 10 mg/kg Q4W + MTX (see Supplementary Figure 14).

### 3.2. Discussion

This study aimed to compare the efficacy and tolerability at 24 weeks of oral tofacitinib 5 mg and 10 mg BID either as monotherapy or combined with MTX or other DMARDs relative to biologic treatments for nonbiologic DMARD-IR RA patients. It should be noted that 24 weeks is a relatively short time frame, especially when assessing long-term efficacy and safety. However, the majority of studies assessed outcomes at 12 and/or 24 weeks and studies assessing long-term efficacy and safety are currently lacking in the literature. The currently available RCTs for tofacitinib provide direct treatment effect estimates relative to placebo and adalimumab.

Since many biologic DMARDs are used to treat DMARD-IR RA patients, it is difficult to completely understand the relative clinical value of tofacitinib by focusing exclusively on the clinical trials of this new oral agent. Therefore, we integrated currently available RCT evidence for competing interventions by performing NMAs to obtain comparative effectiveness estimates of tofacitinib relative to all biologics licensed or seeking approval for RA treatment.

Both as monotherapy and in combination with MTX, tofacitinib 5 mg and 10 mg BID showed comparable ACR20/50/70 responses and physical function improvements to the other available monotherapies. Based on the synthesis of the evidence available for combination biologic therapies, tofacitinib 5 mg and 10 mg BID in combination with DMARDs or MTX were found to be mostly comparable to other combination therapies in terms of efficacy based on ACR20/50/70 criteria and discontinuation due to adverse events.

Meta-analyses are accepted techniques to combine results of multiple RCTs concerning the same pairwise comparisons. In general, the same assumptions apply for an NMA as for a traditional meta-analysis. Patient randomization holds within trials but does not hold across trials; therefore, we can only use relative treatment effects (i.e., log ORs of ACR response or differences in HAQ-DI improvements) in the (network) meta-analysis. For example, it is incorrect to compare ACR20 response observed with one biologic in one trial with ACR20 response with the same or another treatment in another trial. This is because part of the observed response can be attributed to drug efficacy, but another part is due to study and patient characteristics that differ across trials. By using study-specific relative treatment effects as the unit of analysis, any differences in prognostic study and patient characteristics across studies are accounted for and cannot bias the results [24–26]. However, the possibility of study and patient characteristic differences across studies as modifiers of the relative treatment effects exists and remains a source of heterogeneity in studies comparing the same interventions and a source of bias in indirect (or mixed) treatment comparisons [25].

The consistency between direct and indirect estimates for a particular pairwise comparison can potentially be assessed in an evidence network comprised of closed loops. Corresponding mixed treatment estimates are biased in the presence of inconsistency [24–26]. However, in the evidence network of the current study, the closed loops reflected primarily three- or four-arm trials rather than a sequence of two-arm RCTs concerning multiple different comparisons. Therefore, any evaluation of inconsistency between direct and indirect estimates will be very limited and cannot help identify where the NMA might be biased. This leaves only a comparison of study design, patient characteristics, and baseline risk to potentially identify bias. Since the studies did not show differences in patient demographics or baseline components of disease activity, these as sources of bias can be excluded. Patient characteristics were similar across studies.

Differences in discontinuation rates, which may be influenced by protocol requirements for handling nonresponders, can also be a source of bias. In the current analysis, biologics in combination with MTX (or sulfasalazine or multiple DMARDs) and as monotherapy were evaluated simultaneously as part of one network of RCTs. One advantage of this is that all treatment arms of studies that compare an agent plus MTX versus the same agent without MTX can be incorporated [38–41] and that agents combined with MTX can be indirectly compared to agents without MTX.

In recent years, several NMAs of biologic treatments for RA have been published [42–49]. Overall, the findings are comparable despite differences in methodology. Some analyses suggest that certolizumab pegol might be more efficacious than other TNF inhibitors [44, 49], whereas the additional analysis presented here indicates that this might be due to the low placebo response in the certolizumab trials rather than greater absolute efficacy of certolizumab pegol. Some published NMAs only focus on combination therapy, whereas others combine data from both monotherapy and combination therapy studies, either ignoring the effect of MTX or explicitly acknowledging the effect of MTX in a metaregression model [45]. The primary assumption behind such analyses is that the effect of MTX is the same for all biologics, which is contradicted by the analyses presented here.

Our NMA also compared discontinuation rates due to adverse events across interventions. The primary reason for using discontinuation rates instead of specific adverse events rates was inconsistent reporting of adverse events. A limitation of this analysis is the relatively short follow-up reported in RCTs, especially for the tofacitinib studies, so any differences between interventions must be interpreted with caution. The tofacitinib with adalimumab trial showed a comparable incidence of overall adverse events and a small but numerically higher rate of severe infections with tofacitinib compared with adalimumab [21].

## 4. Conclusion

Based on currently available RCT evidence, it can be concluded that oral tofacitinib as 5 or 10 mg BID monotherapy has comparable efficacy to currently available biologic agents used for nonbiologic DMARD-IR RA patients in terms of improvements in signs and symptoms and physical function. Based on the synthesis of the evidence available for combination biologic therapies, tofacitinib 5 mg BID and tofacitinib 10 mg BID in combination with DMARDs or MTX were found to be mostly comparable to other combination therapies in terms of efficacy. Rates of discontinuation from the trials due to adverse events appear comparable for all monotherapies. However, longer-term follow-up data are required to further understand the benefit-risk profile of tofacitinib relative to other combination therapies.

## Supplementary Material

Supplementary Table 1 provides an overview of the patient characteristics across all the identified RCTs, Supplementary Figure 1. ACR20 response at 24 weeks (monotherapy) - Odds ratios and 95% CIs for TOF 5 mg and TOF 10 mg versus other treatments, as obtained with random effects NMA, Supplementary Figure 2. ACR50 response at 24 weeks (monotherapy) – Odds ratios and 95% CIs for TOF 5 mg and TOF 10 mg versus other treatments, as obtained with random effects NMA, Supplementary Figure 3. ACR70 response at 24 weeks (monotherapy) – Odds ratios and 95% CIs for TOF 5 mg and TOF 10 mg versus other treatments, as obtained with random effects NMA, Supplementary Figure 4. Withdrawals due to adverse events (Monotherapy) – Odds ratios and 95% CIs for TOF 5 mg and TOF 10 mg versus other treatments, as obtained with random effects NMA, Supplementary Figure 5. ACR20 response at 24 weeks (combination therapy) - Odds ratios and 95% Cls for TOF 5 mg + DMARDs and TOF 10 mg + DMARDs versus other treatments, as obtained with random effects NMA, Supplementary Figure 6. ACR50 response at 24 weeks (combination therapy) - Odds ratios and 95% Cls for TOF 5 mg + DMARDs and TOF 10 mg + DMARDs versus other treatments, as obtained with random effects NMA, Supplementary Figure 7. ACR70 response at 24 weeks (combination therapy) - Odds ratios and 95% Cls for TOF 5 mg + DMARDs and TOF 10 mg + DMARDs versus other treatments, as obtained with random effects NMA, Supplementary Figure 8. HAQ-DI at 24 weeks (Combination therapies) Modelled change from baseline and 95% CIs for all treatments, as obtained with random effects NMA, Supplementary Figure 9. Withdrawals due to adverse events (Combination therapy) - Odds ratios and 95% Cls for TOF 5 mg + DMARDs and TOF 10 mg + DMARDs versus other treatments, as obtained with random effects NMA, Supplementary Figure 10. ACR20 response at 24 weeks (MTX combination therapy) – Odds ratios and 95% CIs for TOF 5 mg + MTX and TOF 10 mg + MTX versus other treatments, as obtained with random effects NMA, Supplementary Figure 11. ACR50 response at 24 weeks (MTX combination therapy) – Odds ratios and 95% CIs for TOF 5 mg + MTX and TOF 10 mg + MTX versus other treatments, as obtained with random effects NMA, Supplementary Figure 12. ACR70 response at 24 weeks (MTX combination therapy) – Odds ratios and 95% CIs for TOF 5 mg + MTX and TOF 10 mg + MTX versus other treatments, as obtained with random effects NMA, Supplementary Figure 13. HAQ-DI at 24 weeks (MTX combination therapies) - Modelled change from baseline and 95% CIs for all treatments, as obtained with random effects NMA, Supplementary Figure 14. Withdrawals due to adverse events (MTX combination therapy) – Odds ratios and 95% CIs for TOF 5 mg + MTX and TOF 10 mg + MTX versus other treatments, as obtained with random effects NMA.

## Figures and Tables

**Table 1:** Results for monotherapy as obtained with random-effects network meta-analysis at 24 weeks: TOF 5 mg BID and TOF 10 mg BID versus treatment.

Treatment (monotherapy)	ACR20 OR (95% CI)^*∗*^	ACR50 OR (95% CI)^*∗*^	ACR70 OR (95% CI)^*∗*^	Withdrawals due to AEs OR (95% CI)^*∗∗*^	ACR20 OR (95% CI)^*∗∗∗*^	ACR50 OR (95% CI)^*∗∗∗*^	ACR70 OR (95% CI)^*∗∗∗*^	Withdrawals due to AEs OR (95% CI)^*∗∗∗∗*^
	TOF 5 mg BID versus other treatments				TOF 10 mg BID versus other treatments			
TOF 5 mg BID	NA	NA	NA	NA	1.75 (0.47, 6.68)^##^	1.32 (0.33, 5.43)^##^	1.72 (0.33, 10.17)^##^	2.42 (0.30, 16.27)^##^
TOF 10 mg BID	0.57 (0.15, 2.13)^##^	0.76 (0.18, 3.02)^##^	0.58 (0.10, 2.99)^##^	0.41 (0.06, 3.35)^##^	NA	NA	NA	NA
TCZ 8 mg/kg Q4W	0.54 (0.03, 10.36)^##^	0.69 (0.04, 15.01)^##^	0.24 (0.01, 13.81)^##^	0.06 (0.00, 1.48)^##^	0.95 (0.06, 17.60)^##^	0.92 (0.05, 20.57)^##^	0.42 (0.01, 24.62)^##^	0.15 (0.01, 2.74)^##^
CZP 400 mg Q4W	0.43 (0.04, 4.19)^##^	0.63 (0.06, 8.63)^##^	0.21 (0.00, 8.31)^##^	0.13 (0.01, 3.89)^##^	0.75 (0.07, 7.53)^##^	0.84 (0.08, 11.60)^##^	0.38 (0.00, 14.99)^##^	0.33 (0.01, 7.29)^##^
ETN 25 mg BIW	0.31 (0.03, 3.25)^##^	0.38 (0.03, 5.17)^##^	0.24 (0.00, 6.32)^##^	0.61 (0.02, 20.95)^##^	0.55 (0.05, 5.69)^##^	0.51 (0.04, 6.69)^##^	0.42 (0.01, 11.53)^##^	1.47 (0.06, 41.85)^##^
ADA 40 mg QW	0.76 (0.08, 7.30)^##^	0.83 (0.08, 9.62)^##^	0.32 (0.01, 7.84)^##^	0.11 (0.01, 1.58)^##^	1.34 (0.14, 13.42)^##^	1.10 (0.11, 12.91)^##^	0.57 (0.02, 14.64)^##^	0.26 (0.02, 2.90)^##^
ADA 40 mg Q2W	1.03 (0.11, 10.06)^##^	1.60 (0.14, 18.27)^##^	0.53 (0.02, 13.52)^##^	0.05 (0.01, 0.51)^###^	1.81 (0.19, 17.41)^##^	2.10 (0.21, 23.35)^##^	0.92 (0.04, 24.17)^##^	0.13 (0.01, 0.93)^###^
PLBO	3.70 (0.90, 13.16)^##^	5.06 (1.25, 20.37)^###^	4.36 (0.70, 24.15)^##^	0.38 (0.06, 2.88)^##^	6.47 (1.61, 23.13)^###^	6.69 (1.73, 27.65)^###^	7.60 (1.33, 44.03)^###^	0.92 (0.15, 5.27)^##^

^##^Comparable;  ^###^more effective.

^**∗**^OR > 1 favors TOF 5 mg;  ^**∗∗**^OR < 1 favors TOF 5 mg;  ^**∗∗∗**^OR > 1 favors TOF 10 mg;  ^**∗∗∗∗**^OR < 1 favors TOF 10 mg.

**Table 2:** Results for combination therapy as obtained with random-effects network meta-analysis at 24 weeks: TOF 5 mg BID + DMARDs and TOF 10 mg BID + DMARDs versus treatment.

Treatment (+ DMARDs)	ACR20 OR (95% CI)^*∗*^	ACR50 OR (95% CI)^*∗*^	ACR70 OR (95% CI)^*∗*^	Withdrawals due to AEs OR (95% CI)^*∗∗*^	ACR20 OR (95% CI)^*∗∗∗*^	ACR50 OR (95% CI)^*∗∗∗*^	ACR70 OR (95% CI)^*∗∗∗*^	Withdrawals due to AEs OR (95% CI)^*∗∗∗∗*^

	TOF 5 mg BID + DMARDs versus other treatments				TOF 10 mg BID + DMARDs versus other treatments			

TOF 5 mg BID	NA	NA	NA	NA	1.28 (0.71, 2.33)^##^	1.25 (0.63, 2.45)^##^	1.32 (0.74, 2.21)^##^	1.00 (0.63, 1.62)^##^
TOF 10 mg BID	0.78 (0.43, 1.41)^##^	0.80 (0.41, 1.58)^##^	0.76 (0.45, 1.35)^##^	1.00 (0.62, 1.59)^##^	NA	NA	NA	NA
BAR 4 mg QD	0.89 (0.31, 2.51)^##^	1.01 (0.33, 2.95)^##^	1.14 (0.36, 3.06)^##^	NA	1.15 (0.40, 3.22)^##^	1.26 (0.41, 3.71)^##^	1.50 (0.45, 3.92)^##^	NA
BAR 2 mg QD	1.27 (0.45, 3.62)^##^	1.77 (0.58, 5.44)^##^	1.53 (0.52, 4.27)^##^	NA	1.64 (0.58, 4.65)^##^	2.21 (0.72, 6.82)^##^	2.01 (0.67, 5.48)^##^	NA
GLB 50 mg Q4W	0.75 (0.19, 2.94)^##^	0.98 (0.29, 3.22)^##^	1.35 (0.33, 5.13)^##^	3.10 (0.79, 13.40)^##^	0.96 (0.25, 3.74)^##^	1.23 (0.36, 4.04)^##^	1.78 (0.42, 6.60)^##^	3.11 (0.81, 13.48)^##^
TCZ 162 mg Q4W	0.91 (0.23, 3.70)^##^	0.95 (0.23, 3.89)^##^	0.84 (0.24, 2.59)^##^	1.37 (0.54, 3.51)^##^	1.17 (0.29, 4.79)^##^	1.19 (0.28, 4.89)^##^	1.10 (0.31, 3.29)^##^	1.38 (0.44, 7.45)^##^
TCZ 8 mg/kg Q4W	0.83 (0.37, 1.88)^##^	0.90 (0.37, 2.21)^##^	0.71 (0.28, 1.61)^##^	0.97 (0.48, 2.01)^##^	1.06 (0.47, 2.41)^##^	1.13 (0.46, 2.76)^##^	0.93 (0.37, 2.09)^##^	0.97 (0.48, 2.04)^##^
CZP 400 mg Q4W	0.99 (0.25, 3.84)^##^	1.24 (0.25, 5.84)^##^	59.16 (2.70, +infty)^###^	1.83 (0.44, 7.34)^##^	1.27 (0.33, 4.96)^##^	1.56 (0.32, 7.32)^##^	77.40 (3.53, +infty)^###^	1.83 (0.44, 7.45)^##^

	TOF 5 mg				TOF 10 mg			

IFX 3 mg/kg Q8W	1.37 (0.50, 3.77)^##^	1.56 (0.44, 5.58)^##^	1.48 (0.48, 4.34)^##^	1.00 (0.35, 2.82)^##^	1.76 (0.64, 4.85)^##^	1.96 (0.55, 6.97)^##^	1.95 (0.62, 5.57)^##^	1.00 (0.35, 2.85)^##^
ABT 125 mg QW	1.07 (0.37, 3.03)^##^	1.00 (0.33, 2.94)^##^	1.42 (0.53, 3.34)^##^	2.69 (1.12, 6.85)^#^	1.37 (0.47, 3.91)^##^	1.25 (0.41, 3.67)^##^	1.86 (0.68, 4.28)^##^	2.70 (1.13, 6.94)^#^
ABT 10 mg/kg Q4W	1.42 (0.61, 3.29)^##^	1.16 (0.46, 2.90)^##^	1.60 (0.65, 3.57)^##^	1.84 (0.89, 3.98)^##^	1.82 (0.78, 4.21)^##^	1.46 (0.57, 3.65)^##^	2.10 (0.83, 4.61)^##^	1.84 (0.89, 4.03)^##^
ETN 50 mg QW	1.36 (0.49, 3.76)^##^	1.52 (0.52, 4.47)^##^	1.56 (0.57, 4.06)^##^	3.17 (0.63, 15.64)^##^	1.74 (0.63, 4.84)^##^	1.91 (0.65, 5.62)^##^	2.04 (0.73, 5.22)^##^	3.18 (0.63, 16.07)^##^
ETN 25 mg BIW	0.74 (0.28, 1.90)^##^	1.01 (0.33, 2.74)^##^	1.40 (0.44, 3.47)^##^	2.35 (0.94, 5.50)^##^	0.96 (0.36, 2.42)^##^	1.26 (0.41, 3.40)^##^	1.85 (0.56, 4.52)^##^	2.36 (0.95, 5.53)^##^
ADA 40 mg Q2W	0.82 (0.39, 1.69)^##^	0.94 (0.43, 1.99)^##^	1.43 (0.68, 2.74)^##^	1.38 (0.72, 2.75)^##^	1.06 (0.50, 2.17)^##^	1.19 (0.53, 2.48)^##^	1.87 (0.87, 3.56)^##^	1.38 (0.72, 2.78)^##^
PLBO	2.88 (1.61, 5.17)^###^	4.43 (2.22, 8.78)^###^	6.33 (3.21, 12.14)^###^	2.04 (1.18, 3.63)^#^	3.70 (2.07, 6.61)^###^	5.55 (2.76, 10.97)^###^	8.29 (4.13, 15.75)^###^	2.06 (1.19, 3.66)^#^

^#^Less effective;  ^##^comparable;  ^###^more effective.

^*∗*^OR > 1 favors TOF 5 mg;  ^*∗∗*^OR < 1 favors TOF 5 mg;  ^*∗∗∗*^OR > 1 favors TOF 10 mg;  ^*∗∗∗∗*^OR < 1 favors TOF 10 mg.

**Table 3:** Results for MTX combination therapy as obtained with random-effects network meta-analysis at 24 weeks: TOF 5 mg BID + MTX and TOF 10 mg BID + MTX versus treatment.

Treatment (+ MTX)	ACR20 OR (95% CI)^*∗*^	ACR50 OR (95% CI)^*∗*^	ACR70 OR (95% CI)^*∗*^	Withdrawals due to AEs OR (95% CI)^*∗∗*^	ACR20 OR (95% CI)^*∗∗∗*^	ACR50 OR (95% CI)^*∗∗∗*^	ACR70 OR (95% CI)^*∗∗∗*^	Withdrawals due to AEs OR (95% CI)^*∗∗∗∗*^
	TOF 5 mg BID + MTX versus other treatments				TOF 10 mg BID + MTX versus other treatments			
TOF 5 mg BID	NA	NA	NA	NA	1.31 (0.57, 3.02)^##^	1.25 (0.59, 2.65)^##^	1.32 (0.74, 2.21)^##^	0.79 (0.41, 1.70)^##^
TOF 10 mg BID	0.76 (0.33, 1.76)^##^	0.80 (0.38, 1.71)^##^	0.76 (0.45, 1.36)^##^	1.26 (0.59, 2.46)^##^	NA	NA	NA	NA
GLB 50 mg Q4W	0.81 (0.15, 4.42)^##^	1.00 (0.26, 3.64)^##^	1.32 (0.32, 4.98)^##^	2.84 (0.63, 13.21)^##^	1.07 (0.20, 5.74)^##^	1.26 (0.33, 4.54)^##^	1.73 (0.41, 6.43)^##^	2.25 (0.51, 10.87)^##^
TCZ 8 mg/kg Q4W	0.86 (0.24, 3.07)^##^	0.89 (0.27, 2.87)^##^	0.71 (0.24, 1.91)^##^	0.86 (0.30, 2.47)^##^	1.12 (0.31, 4.04)^##^	1.11 (0.33, 3.60)^##^	0.93 (0.31, 2.49)^##^	0.68 (0.24, 2.09)^##^
CZP 400 mg Q4W	1.08 (0.20, 5.73)^##^	1.27 (0.23, 6.65)^##^	53.28 (2.66, +infty)^###^	1.68 (0.31, 8.22)^##^	1.42 (0.26, 7.47)^##^	1.60 (0.29, 8.32)^##^	70.27 (3.49, +infty)^###^	1.35 (0.25, 6.79)^##^
IFX 3 mg/kg Q8W	1.48 (0.41, 5.22)^##^	1.58 (0.39, 6.53)^##^	1.46 (0.47, 4.31)^##^	0.94 (0.27, 3.14)^##^	1.94 (0.54, 6.85)^##^	1.99 (0.48, 8.03)^##^	1.92 (0.59, 5.51)^##^	0.75 (0.21, 2.58)^##^
ABT 125 mg QW	1.04 (0.27, 3.94)^##^	0.97 (0.28, 3.21)^##^	1.43 (0.52, 3.31)^##^	2.62 (0.83, 8.59)^##^	1.36 (0.35, 5.15)^##^	1.22 (0.35, 4.01)^##^	1.88 (0.66, 4.27)^##^	2.08 (0.68, 7.22)^##^
ABT 10 mg/kg Q4W	1.50 (0.50, 4.44)^##^	1.17 (0.42, 3.26)^##^	1.59 (0.64, 3.50)^##^	1.83 (0.65, 5.15)^##^	1.96 (0.65, 5.80)^##^	1.47 (0.52, 4.04)^##^	2.10 (0.68, 5.84)^##^	1.45 (0.53, 4.30)^##^
ETN 50 mg QW	1.49 (0.40, 5.39)^##^	1.56 (0.47, 5.18)^##^	1.53 (0.56, 3.99)^##^	2.99 (0.45, 17.55)^##^	1.95 (0.53, 7.01)^##^	1.96 (0.59, 6.41)^##^	2.02 (0.72, 5.11)^##^	2.38 (0.37, 14.70)^##^
ETN 25 mg BIW	1.08 (0.27, 1.95)^##^	1.24 (0.30, 4.11)^##^	1.75 (0.54, 4.50)^##^	2.37 (0.59, 7.31)^##^	1.41 (0.35, 5.31)^##^	1.55 (0.37, 5.12)^##^	2.31 (0.68, 5.84)^##^	1.88 (0.50, 6.08)^##^
ADA 40 mg Q2W	0.74 (0.27, 1.95)^##^	0.87 (0.35, 2.03)^##^	1.46 (0.66, 2.76)^##^	1.31 (0.56, 3.23)^##^	0.97 (0.35, 2.55)^##^	1.09 (0.44, 2.54)^##^	1.92 (0.84, 3.60)^##^	1.04 (0.46, 2.75)^##^
PLBO	3.15 (1.39, 7.05)^###^	4.55 (2.11, 9.66)^###^	6.19 (3.15, 12.04)^###^	1.89 (0.86, 3.87)^##^	4.11 (1.82, 9.26)^###^	5.68 (2.65, 11.98)^###^	8.16 (4.04, 15.65)^###^	1.51 (0.71, 3.28)^##^

^##^Comparable;  ^###^more effective.

^*∗*^OR > 1 favors TOF 5 mg; ^*∗∗*^OR < 1 favors TOF 5 mg; ^*∗∗∗*^OR > 1 favors TOF 10 mg; ^*∗∗∗∗*^OR < 1 favors TOF 10 mg.

**Table 4:** 

Study name	Trial/author, year	Was randomization carried out appropriately?	Was the concealment of treatment allocation adequate?	Were the care providers, participants, and outcome assessors blind to treatment allocation?	Were the groups similar at the outset of the study in terms of prognostic factors, for example, severity of disease?	Were there any unexpected imbalances in drop-outs between groups?	Is there any evidence to suggest that the authors measured more outcomes than they reported?	Did the analysis include an intention-to-treat analysis? If so, was this appropriate and were appropriate methods used to account for missing data?
BREVACTA	Kivitz, 2014; Olech, 2013 (abstract)	Yes	Yes	Yes	Yes	No	No	Yes
TOWARD	Genovese, 2008	Unclear	Unclear	Yes	Yes	No	No	Yes
REALISTIC	Weinblatt, 2012	Yes	Yes	Yes	Yes	No	No	Yes
ROSE	Yazici, 2012	Unclear	Unclear	Yes	Yes	No	No	Yes
ACQUIRE	Genovese, 2011	Yes	Unclear	Yes	Yes	No	No	Yes
AMPLE	Weinblatt, 2013	Unclear	Unclear	No	Yes	No	No	Yes
AUGUST II	van Vollenhoven, 2011	Yes	Yes	Yes	Yes	No	No	Yes
ACT–RAY	Dougados, 2013	Yes	Yes	Yes	Yes	No	No	Yes
ADACTA	Gabay, 2013	Yes	Yes	Yes	Yes	No	No	Yes
ABT-Kremer 1	Kremer, 2003; Kremer, 2005	Yes	Yes	Yes	Yes	No	No	Unclear
ABT-Kremer 2	Kremer, 2006; Russell, 2007	Yes	Yes	Yes	Yes	No	No	Yes
ATTEST	Schiff, 2008	Yes	Unclear	Yes	Yes	No	No	Yes
ADA–Van de Putte 2	Van de Putte, 2004	Yes	Yes	Yes	Yes	No	No	Yes
ADA–Van de Putte 1	Van de Putte, 2003	Unclear	Unclear	Yes	Yes	No	No	Yes
ARMADA	Weinblatt, 2003	Yes	Yes	Yes	Yes	No	No	Yes
ADA–Keystone	Keystone, 2004	Unclear	Unclear	Yes	Yes	No	No	Yes
FAST4WARD	Fleischmann, 2009	Yes	Yes	Yes	Yes	No	No	Yes
RAPID 1	Keystone, 2008; Strand, 2009	Unclear	Unclear	Yes	Yes	No	No	Yes
RAPID 2	Smolen, 2009; Strand, 2011	Unclear	Unclear	Yes	Yes	No	No	Yes
NCT00544154	Choy, 2012	Yes	Yes	Yes	Yes	No	No	Yes
ETN–Mathias/Moreland	Moreland, 1999; Mathias, 2000	Yes	Yes	Yes	Yes	No	No	Yes
ETN–Weinblatt	Weinblatt, 1999	Yes	Yes	Yes	Yes	No	No	Unclear
ADORE	Van Riel, 2006; van Riel, 2008	Unclear	Unclear	No	Yes	No	No	Yes
ETN–Combe	Combe, 2006; Combe, 2009	Unclear	Unclear	Yes	Yes	No	No	Yes
GLB–Kay	Kay, 2008	Yes	Yes	Yes	Yes	No	No	Unclear
GO–FORWARD	Keystone, 2009; Genovese, 2012	Yes	Yes	Yes	Yes	No	No	Unclear
ATTRACT	Lipsky, 2000; Maini, 1999	Yes	Yes	Yes	Yes	No	No	Yes
START	Westhovens, 2006	Unclear	Unclear	Yes	Yes	No	No	Yes
CHARISMA	Maini, 2006	Yes	Yes	Yes	Yes	No	No	Yes
OPTION	Smolen, 2008	Yes	Yes	Yes	Yes	No	No	Yes
LITHE	Kremer, 2011; Fleischmann, 2013	Yes	Yes	Yes	Yes	No	No	Yes
ORAL-Solo (Pfizer A3921045)	Fleishmann, 2012	Yes	Yes	Yes	Yes	No	No	Yes
TOFA monotherapy (Pfizer A3921035)	Fleischmann, 2012	Unclear	Unclear	Yes	Yes	No	No	Unclear
ORAL-Standard (Pfizer A3921064)	van Vollenhoven, 2012	Yes	Yes	Unclear	Yes	No	No	Unclear
TOFA+MTX–Kremer (Pfizer A3921025)	Kremer, 2012	Unclear	Unclear	Yes	Yes	No	No	Unclear
ORAL-Scan (Pfizer A3921044)	Van der Heijde, 2013; van der Heijde, 2012; van der Heijde, 2011	Yes	Yes	Yes	Yes	No	No	Unclear

## Appendix

### A. Search Strategy

1. “randomized controlled trial”.pt.
2. (random$ or placebo$ or single blind$ or double blind$ or triple blind$).ti,ab.
3. (retraction of publication or retracted publication).pt.
4. (1) or (2) or (3)
5. (animals not humans).sh.
6. ((comment or editorial or meta-analysis or practicD-guideline or review or letter or journal correspondence) not “randomized controlled trial”).pt.
7. (random sampl$ or random digit$ or random effect$ or random survey or random regression).ti,ab. not “randomized controlled trial”.pt.
8. (5) or (6) or (7)
9. (4) not (8)
10. (random$ or placebo$ or single blind$ or double blind$ or triple blind$).ti,ab.
11. RETRACTED ARTICLE/
12. (10) or (11)
13. (animal$ not human$).sh,hw.
14. (book or conference paper or editorial or letter or review).pt. not exp randomized controlled trial/
15. (random sampl$ or random digit$ or random effect$ or random survey or random regression).ti,ab. not exp randomized controlled trial/
16. (13) or (14) or (15)
17. (12) not (16)
18. (9) or (17)
19. Arthritis, Rheumatoid/
20. rheumatoid arthritis.ti,ab.
21. (19) or (20)
22. (tumour necrosis factor or tumour necrosis factor inhibitor or tumour necrosis factor blocker or tumour necrosis factor receptor or anti- tumour necrosis factor or TNF or anti-TNF).ti,ab.
23. (biologic or biological).ti,ab
24. (janus kinase or JAK or jakinibs or janus associated kinase).ti,ab.
25. (abatacept or Orencia or CTLA-4Ig).ti,ab.
26. (adalimumab or Humira).ti,ab.
27. (anakinra or Kineret).ti,ab.
28. (baricitinib or LY3009104).ti,ab.
29. (certolizumab or Cimzia or CDP870).ti,ab.
30. (etanercept or Enbrel).ti,ab.
31. (golimumab or Simponi or CNTO 148).ti,ab.
32. (infliximab or Remicade).ti,ab.
33. (rituximab or Rituxan or Mabthera).ti,ab.
34. (tocilizumab or Actemra or RoActemra).ti,ab.
35. (tofacitinib or tasaocitinib or CP-690550).ti,ab.
36. (secukinumab or AIN-457).ti,ab.
37. (sarilumab or SAR153191 or REGN88).ti,ab.
38. (sirukumab or CNTO 136).ti,ab.
39. or/(22)–(38)
40. (18) and (21) and (39).

### B. Quality Assessment Checklist and Results

See Table 4.

## Additional Points

(i) The efficacy and safety of tofacitinib, an oral Janus kinase inhibitor for the treatment of rheumatoid arthritis (RA), were compared to other biologic treatments in patients with moderate-to-severe RA. (ii) Conventional DMARDS were not developed based on a specific mechanism of action. Newer, targeted synthetic (ts) DMARDs were developed to target specific components of the inflammation response, differentiating them as a class. (iii) The orally bioavailable, synthetic, small-molecule Janus kinase (JAK) inhibitor tofacitinib is one example of a tsDMARD. (iv) The analysis presented evaluates tofacitinib in patients who are intolerant or experience moderate/high disease activity despite traditional, nonbiologic DMARDs. (v) NMA models combining both direct and indirect evidences for each of the endpoints of interest were performed. (vi) In most cases, tofacitinib had similar efficacy and discontinuation rates due to adverse events compared to biologic DMARDs in patients with moderate-to-severe RA.
