# Supplementary material for: Tofacitinib versus Biologic Treatments in Moderate-to-Severe Rheumatoid Arthritis Patients Who Have Had an Inadequate Response to Nonbiologic DMARDs: Systematic Literature Review and Network Meta-Analysis
Source: Int J Rheumatol. 2017 Mar 9;2017:8417249. doi: 10.1155/2017/8417249 (PMC5362710; doi:10.1155/2017/8417249)

Supplementary Table 1. Patient Characteristics

| **Study** | **Intervention(s)** | **Patients (n)** | **Female (%)** | **Age (years)** | **Disease duration (years)** | **SJC (number of joints)** | **TJC (number of joints)** | **ESR (mm/h)** | **CRP (mg/L)** |
| --- | --- | --- | --- | --- | --- | --- | --- | --- | --- |
| ABT-Kremer 1 | PLBO | 119 | 66 | 54.7 | 8.9 | 21.8 | 29.2 | NR | 3.2 mg/dl |
|  | ABT 2 mg/kg | 105 | 63 | 54.4 | 9.7 | 20.2 | 28.2 | NR | 3.2 mg/dl |
|  | ABT 10 mg/kg | 115 | 75 | 55.8 | 9.7 | 21.3 | 30.8 | NR | 2.9 mg/dl |
| ACQUIRE | ABT (SC) | 736 | 84.4 | 49.9 | 7.6 | 20.4 | 30.1 | NR | 2.6 mg/dl |
|  | ABT (IV) | 721 | 80.4 | 50.1 | 7.7 | 19.4 | 29.1 | NR | 2.7 mg/dl |
| ACT-RAY | TCZ + MTX | 277 | 81.9 | 53 | 8.2 | 14.4 | 25.8 | NR | NR |
|  | TCZ + PLBO | 276 | 78.6 | 53.6 | 8.3 | 15.3 | 26.6 | NR | NR |
| ADACTA | TCZ | 163 | 82.0 | 54.4 | 6.3 | 11.3 | 15.9 | 45.5 | 2.6 x10^-2 g/l |
|  | ADA | 162 | 89.0 | 53.3 | 7.3 | 12.4 | 16.5 | 50.5 | 2.5 x10^-2 g/l |
| ADA-Van de Putte 1 | PLBO | 70 | 81.0 | 50.2 | 9.4 | 20.2 | 30.9 | 53.6 | 63.0 |
|  | ADA | 70 | 81.0 | 52.6 | 10.0 | 18.7 | 31.0 | 51.7 | 56.0 |
|  | ADA | 72 | 69.0 | 53.2 | 10.1 | 19.6 | 32.5 | 50.7 | 60.0 |
|  | ADA | 72 | 85.0 | 53.7 | 10.4 | 19.6 | 31.7 | 52.9 | 57.0 |
| ADA-Van de Putte 2 | ADA | 106 | 79.0 | 53.1 | 9.3 | 19.6 | 33.9 | 52.8 | 52.4 |
|  | ADA | 113 | 79.6 | 52.7 | 10.6 | 20.5 | 33.7 | 55.8 | 52.6 |
|  | ADA | 112 | 72.3 | 54.4 | 11.3 | 19.8 | 35.3 | 51.5 | 47.2 |
|  | PLBO | 110 | 77.3 | 53.5 | 11.6 | 19.8 | 35.5 | 56.1 | 57.0 |
|  | ADA | 103 | 78.6 | 51.8 | 11.9 | 19.3 | 33.8 | 51.1 | 49.3 |
| ADORE | ETN + MTX | 155 | 76.8 | 54 | 9.8 | 11.9 | 14.7 | 36.7 | NR |
|  | ETN + PLBO | 159 | 79.2 | 53 | 10.0 | 11.2 | 14.6 | 33.2 | NR |
| AIM | ABT | 433 | 77.8 | 51.5 | 8.5 | 21.4 | 31.0 | NR | 33.0 |
|  | PLBO | 219 | 81.7 | 50.4 | 8.9 | 22.1 | 32.3 | NR | 28.0 |
| AMPLE | ADA | 328 | 82.3 | 51 | 1.7 | 15.9 | 26.3 | NR | 1.5 mg/dl |
|  | ABT | 318 | 81.4 | 51.4 | 1.9 | 15.8 | 25.4 | NR | 1.6 mg/dl |
| ANA-Cohen 1 | ANA | 59 | 84.7 | 49 | 6.5 | 17.6 | 22.0 | 37.1 | 1.6 mg/dl |
|  | PLBO | 74 | 85.1 | 53 | 7.8 | 18.4 | 28.1 | 36.2 | 2.0 mg/dl |
| ANA-Cohen 2 | PLBO | 250 | 75.0 | 57 | 10.0 | 20.0 | 24.5 | 42.9 | 26.0 |
|  | ANA | 251 | 79.0 | 56 | 11.0 | 20.1 | 26.8 | 41.5 | 27.0 |
| ARMADA | PLBO | 62 | 82.3 | 56 | 11.1 | 16.9 | 28.7 | NR | 3.1 mg/dl |
|  | ADA | 67 | 74.6 | 57.2 | 12.2 | 17.3 | 28.0 | NR | 2.1 mg/dl |
|  | ADA | 73 | 75.3 | 55.5 | 12.8 | 17.0 | 30.3 | NR | 2.8 mg/dl |
|  | ADA | 69 | 75.4 | 53.5 | 13.1 | 17.6 | 28.5 | NR | 2.8 mg/dl |
| ASSET | ABT | 27 | 59.3 | 51.7 | 2.1 | 11.3 | 12.9 | NR | 13.6 |
|  | PLBO | 23 | 69.6 | 52.5 | 2.4 | 8.5 | 13.3 | NR | 16.6 |
| ASSURE | PLBO | 418 | 83.7 | 52 | 9.5 | NR | NR | NR | 2.1 mg/dl |
|  | ABT | 856 | 83.1 | 52.2 | 9.5 | NR | NR | NR | 1.9 mg/dl |
| ATTEST | IFX | 165 | 82.0 | 49.1 | 7.3 | 20.3 | 31.7 | 47.8 | 3.3 mg/dl |
|  | ABT | 156 | 83.0 | 49 | 7.9 | 21.3 | 31.6 | 49.4 | 3.1 mg/dl |
|  | PLBO | 110 | 87.0 | 49.4 | 8.4 | 20.1 | 30.3 | 47.0 | 2.7 mg/dl |
| ATTRACT | IFX | 86 | 77.0 | 52 | 9.0 | 21.0 | 31.0 (median) | 52.0 | 3.5 mg/dl |
|  | IFX | 86 | 81.0 | 54 | 10.0 | 22.0 | 32.0 (median) | 49.0 | 3.9 mg/dl |
|  | PLBO | 88 | 80.0 | 51 | 11.0 | 21.0 | 24.0 (median) | 49.0 | 4.0 mg/dl |
|  | IFX | 87 | 77.0 | 54 | 11.0 | 23.0 | 30.0 (median) | 50.0 | 3.3 mg/dl |
|  | IFX | 81 | 73.0 | 52 | 12.0 | 24.0 | 35.0 (median) | 49.0 | 4.2 mg/dl |
| AUGUST II | PLBO | 76 | 84.0 | 54 | 8.4 | 16.4 | 24.3 | 39.3 | 16.5 |
|  | ADA | 79 | 81.0 | 53 | 8.8 | 16.2 | 27.8 | 41.7 | 16.6 |
| BREVACTA | PLBO | 218 | 82.6 | 52 | 11.1 | 17.6 | 27.5 | 49.4 | 1.9 mg/dl |
|  | TCZ | 438 | 85.8 | 52.1 | 11.1 | 17.5 | 28.1 | 50.9 | 2.0 mg/dl |
| CHARISMA | TCZ + MTX | 49 | NR | 50.2 | 0.65 | 11.0 | 13.0 | 40.0 | 31.0 |
|  | TCZ + PLBO | 53 | NR | 52.2 | 0.77 | 11.0 | 15.0 | 39.0 | 26.0 |
|  | TCZ + PLBO | 52 | NR | 50.1 | 0.77 | 12.0 | 15.0 | 39.0 | 22.0 |
|  | TCZ + MTX | 52 | NR | 49.2 | 0.78 | 11.0 | 15.0 | 45.0 | 28.0 |
|  | TCZ + PLBO | 54 | NR | 49.3 | 0.82 | 11.0 | 15.0 | 41.0 | 19.0 |
|  | TCZ + MTX | 50 | NR | 50.1 | 0.89 | 11.0 | 15.0 | 39.0 | 24.0 |
|  | PLBO + MTX | 49 | NR | 50.9 | 0.94 | 12.0 | 16.0 | 43.0 | 32.0 |
| CSP 551 RACAT | ETN | 175 | 48.6 | 56 | 4.90 | 11.3 | 13.3 | 29.7 | NR |
|  | SSZ + HCQ | 178 | 43.3 | 57.8 | 5.50 | 11.1 | 13.4 | 27.4 | NR |
| DE019 | PLBO | 200 | 73.0 | 56.1 | 10.9 | 19.0 | 28.1 | NR | 1.8 mg/dl |
|  | ADA | 207 | 76.3 | 56.1 | 11.0 | 19.3 | 27.3 | NR | 1.8 mg/dl |
|  | ADA | 212 | 75.5 | 57.3 | 11.0 | 19.6 | 27.9 | NR | 1.4 mg/dl |
| ETN-Combe | PLBO + SSZ | 50 | 82.0 | 53.3 | 5.6 | 18.7 | 31.3 | 37.2 | 11.6 (median) |
|  | ETN + SSZ | 101 | 80.2 | 50.6 | 6.5 | 19.4 | 31.3 | 39.0 | 11.6 (median) |
|  | ETN +PLBO | 103 | 78.6 | 51.3 | 7.1 | 19.1 | 29.7 | 37.4 | 14.3 (median) |
| ETN-Johnsen | ETN | 26 | 88.0 | 50.5 (median) | 12.5 (median) | 24.0 | 32.0 | NR | NR |
|  | ETN | 51 | 84.0 | 55 (median) | 15.0 (median) | 22.0 | 32.0 | NR | NR |
| ETN-Mathias/Moreland | ETN | 78 | 74.4 | 53 | 11.0 | 25.0 | 33.0 | 35.0 | 4.7 mg/dl |
|  | PLBO | 80 | 76.3 | 51 | 12.0 | 25.0 | 35.0 | 39.0 | 4.1 mg/dl |
|  | ETN | 76 | 84.2 | 53 | 13.0 | 25.0 | 34.0 | 44.0 | 5.3 mg/dl |
| ETN-Weinblatt | PLBO | 30 | 73.0 | 53 | 13.0 | 17.0 (median) | 28.0 (median) | 36.0 (median) | 2.6 mg/dl (median) |
|  | ETN | 59 | 90.0 | 48 | 13.0 | 20.0 (median) | 28.0 (median) | 25.0 (median) | 2.2 mg/dl (median) |
| FAST4WARD | CZP | 111 | 78.4 | 52.7 | 8.7 | 21.2 | 29.6 | 30.9 | 11.6 |
|  | PLBO | 109 | 89.0 | 54.9 | 10.4 | 19.9 | 28.3 | 35.6 | 11.3 |
| GLB-Kay | PLBO | 35 | 74.3 | 52 (median) | 5.6 (median) | 13.0 (median) | 22.0 (median) | NR | 2.0 mg/dl (median) |
|  | GLB | 34 | 76.5 | 57.5 (median) | 6.3 (median) | 20.00 (median) | 32.00 (median) | NR | 1.4 mg/dl (median) |
|  | GLB | 35 | 85.7 | 57 (median) | 8.2 (median) | 14.00 (median) | 28.00 (median) | NR | 2.1 mg/dl (median) |
|  | GLB | 34 | 67.6 | 48 (median) | 8.2 (median) | 14.00 (median) | 28.00 (median) | NR | 1.6 mg/dl (median) |
|  | GLB | 34 | 79.4 | 53.5 (median) | 9.0 (median) | 14.00 (median) | 22.00 (median) | NR | 1.6 mg/dl (median) |
| GO-FORWARD | GLB + MTX | 89 | 80.9 | 50.3 | 7.3 | 13.00 (median) | 26.00 (median) | NR | 1.0 mg/dl (median) |
|  | GLB + PLBO | 133 | 78.9 | 50 | 8.3 | 11.00 (median) | 22.00 (median) | NR | 0.9 mg/dl (median) |
|  | PLBO + MTX | 133 | 82.0 | 51.2 | 8.6 | 12.00 (median) | 21.00 (median) | NR | 0.8 mg/dl (median) |
|  | GLB + MTX | 89 | 80.9 | 50 | 9.0 | 12.00 (median) | 23.00 (median) | NR | 0.9 mg/dl (median) |
| GO-FURTHER | GLB | 395 | 82.5 | 51.9 | 6.9 | 15.0 | 26.4 | NR | 2.8 mg/dl |
|  | PLBO | 197 | 79.7 | 51.4 | 7.0 | 14.8 | 25.9 | NR | 2.2 mg/dl |
| I4V-MC-JADA | BAR | 52 | 71.0 | 53 | 5.3 | 14.8 | 19.9 | 35.4 | 11.4 |
|  | PLBO > BAR | 98 | 87.0 | 49 | 5.4 | 15.8 | 22.2 | 39.9 | 14.0 |
|  | BAR | 49 | 86.0 | 53 | 5.5 | 15.2 | 21.4 | 38.2 | 11.2 |
|  | BAR | 52 | 85.0 | 51 | 5.5 | 17.0 | 23.0 | 36.5 | 12.0 |
|  | BAR | 50 | 82.0 | 53 | 6.6 | 16.1 | 24.4 | 43.3 | 14.3 |
| IFX-Maini | PLBO + MTX | 14 | 71.0 | 48.8 | 7.6 | 17.00 (median) | 28.00 (median) | 50.0 (median) | 5.1 mg/dl (median) |
|  | IFX + PLBO | 14 | 86.0 | 47 | 7.8 | 17.00 (median) | 31.00 (median) | 31.0 (median) | 1.8 mg/dl (median) |
|  | IFX + MTX | 15 | 67.0 | 58.9 | 12.1 | 16.00 (median) | 21.00 (median) | 60.0 (median) | 4.2 mg/dl (median) |
| LITHE | PLBO | 398 | 83.0 | 51.3 | 9.0 | 16.6 | 27.9 | 46.5 | 2.2 mg/dl |
|  | TCZ | 393 | 82.0 | 53.4 | 9.3 | 17.3 | 29.3 | 46.4 | 2.3 mg/dl |
|  | TCZ | 399 | 84.0 | 51.4 | 9.4 | 17.0 | 27.9 | 45.9 | 2.1 mg/dl |
| NCT00361335 | PLBO + MTX | 129 | 79.8 | 50.2 | 7.4 | 16.1 | 28.2 | 41.6 | 1.7 mg/dl |
|  | GLB + PLBO | 128 | 83.6 | 49.9 | 7.4 | 15.7 | 28.1 | 41.7 | 1.8 mg/dl |
|  | GLB + MTX | 129 | 76.7 | 49.7 | 8.1 | 15.5 | 26.8 | 40.9 | 1.8 mg/dl |
|  | GLB + PLBO | 129 | 81.4 | 48.4 | 8.4 | 15.2 | 26.5 | 40.2 | 1.7 mg/dl |
|  | GLB + MTX | 128 | 80.5 | 49.6 | 9.4 | 15.3 | 27.1 | 41.2 | 2.0 mg/dl |
| NCT00544154 | CZP | 126 | 72.2 | 53 | 9.4 | 22.8 | 29.0 | 24.4 | 11.9 |
|  | PLBO | 121 | 66.1 | 55.6 | 9.9 | 22.2 | 31.0 | 25.9 | 13.1 |
| NCT00718718; Part B | SIR | 31 | 74.2 | 52.8 | 6.6 | 12.9 | 24.5 | NR | 2.0 mg/dl |
|  | PLBO > SIR | 30 | 83.3 | 54.1 | 7.7 | 15.1 | 24.2 | NR | 2.0 mg/dl |
|  | SIR | 30 | 90.0 | 53.8 | 8.3 | 14.9 | 22.3 | NR | 2.6 mg/dl |
|  | SIR | 30 | 90.0 | 52 | 9.3 | 16.2 | 29.1 | NR | 2.8 mg/dl |
|  | SIR | 30 | 86.7 | 50.9 | 9.9 | 14.2 | 26.4 | NR | 2.4 mg/dl |
| NCT00848354 | ETN | 281 | 88.3 | 48.4 | 7.9 | 18.2 | 25.1 | 43.2 | 20.7 |
|  | SSZ or HCQ | 142 | 90.1 | 48.6 | 9.0 | 19.3 | 26.2 | 42.8 | 20.8 |
| NCT00928512 | SEC | 41 | 75.6 | 54.7 | 5.9 | 11.7 | 15.3 | NR | 7.5 (median) |
|  | SEC | 54 | 83.3 | 53.3 | 6.7 | 11.1 | 14.6 | NR | 9.5 (median) |
|  | PLBO | 50 | 68.0 | 55 | 6.8 | 10.7 | 14.5 | NR | 11.1 (median) |
|  | SEC | 43 | 81.4 | 57.8 | 7.9 | 10.9 | 14.4 | NR | 10.4 (median) |
|  | SEC | 49 | 77.3 | 54.3 | 8.6 | 11.0 | 13.5 | NR | 16.3 (median) |
| OPTION | TCZ | 213 | 82.0 | 51.4 | 7.4 | 20.0 | 33.2 | 49.2 | 28.0 |
|  | TCZ | 205 | 85.0 | 50.8 | 7.5 | 19.5 | 31.9 | 51.2 | 26.0 |
|  | PLBO | 204 | 78.0 | 50.6 | 7.8 | 20.7 | 32.8 | 49.7 | 24.0 |
| ORAL SYNC (A3921046) | TOFA | 315 | 83.8 | 52.7 | 8.1 | 14.5 | 25.0 | 50.5 | 168.4 nmol/l |
|  | TOFA | 318 | 81.1 | 51.9 | 9.2 | 14.4 | 26.6 | 51.9 | 168.9 nmol/l |
|  | PLBO > TOFA | 79 | 79.7 | 50.8 | 9.5 | 14.6 | 27.2 | 51.0 | 160.8 nmol/l |
|  | PLBO > TOFA | 80 | 75.0 | 53.3 | 10.2 | 13.9 | 21.9 | 49.3 | 157.5 nmol/l |
| ORAL-Scan | PLBO > TOFA | 81 | 80.2 | 53.2 | 8.8 | 14.0 | 23.3 | 47.8 | 12.2 |
|  | TOFA | 321 | 83.8 | 53.7 | 8.9 | 14.1 | 24.1 | 50.1 | 15.5 |
|  | TOFA | 316 | 86.4 | 52 | 9.0 | 14.4 | 23.0 | 50.5 | 17.0 |
|  | PLBO > TOFA | 79 | 91.1 | 52.1 | 9.5 | 14.5 | 22.6 | 54.4 | 15.3 |
| ORAL-Solo | PLBO > TOFA | 122 | 86.1 | 49.7 | 7.7 | 17.3 | 28.9 | 50.9 | 17.8 |
|  | TOFA | 243 | 85.2 | 52.2 | 8.0 | 16.3 | 29.4 | 53.1 | 22.9 |
|  | TOFA | 245 | 88.2 | 52.4 | 8.6 | 17.0 | 29.1 | 52.1 | 19.1 |
| ORAL-Standard | PLBO > TOFA | 56 | 76.8 | 55.5 | 6.9 | 16.9 | 26.6 | 52.7 | 20.3 |
|  | TOFA | 201 | 83.6 | 52.9 | 7.4 | 15.8 | 26.1 | 49.9 | 17.3 |
|  | TOFA | 204 | 85.3 | 53 | 7.6 | 16.7 | 28.5 | 48.6 | 14.9 |
|  | ADA | 204 | 79.4 | 52.5 | 8.1 | 16.4 | 26.7 | 48.5 | 17.5 |
|  | PLBO > TOFA | 52 | 75.0 | 51.9 | 9.0 | 16.4 | 28.1 | 42.9 | 11.6 |
| Pfizer A3921025 | TOFA | 71 | 80.3 | 52 | 9.0 | 14.1 | 21.5 | NR | 18.0 |
|  | TOFA | 74 | 74.3 | 56 | 7.5 | 14.8 | 24.8 | NR | 14.4 |
|  | PLBO | 69 | 81.2 | 53 | 9.2 | 15.7 | 21.6 | NR | 18.9 |
| Pfizer A3921035 | ADA | 53 | 84.9 | 54 | 7.7 | 14.9 | 24.1 | 44.8 | 20.1 |
|  | TOFA | 49 | 87.8 | 54 | 8.1 | 17.4 | 27.1 | 47.4 | 24.5 |
|  | TOFA | 61 | 86.9 | 52 | 8.6 | 16.3 | 25.7 | 43.8 | 16.7 |
|  | PLBO | 59 | 88.1 | 53 | 10.8 | 16.9 | 25.9 | 46.2 | 23.5 |
| RA-BUILD | PLBO | 228 | NR | NR | NR | NR | NR | NR | NR |
|  | BAR | 229 | NR | NR | NR | NR | NR | NR | NR |
|  | BAR | 227 | NR | NR | NR | NR | NR | NR | NR |
| RAPID 1 | CZP | 393 | 82.4 | 51.4 | 6.1 | 21.7 | 30.8 | 43.5 (median) | 16.0 (median) |
|  | PLBO | 199 | 83.9 | 52.2 | 6.2 | 21.2 | 29.8 | 45 (median) | 32.0 (median) |
|  | CZP | 390 | 83.6 | 52.4 | 6.2 | 21.5 | 31.1 | 42.5 (median) | 14.0 (median) |
| RAPID 2 | PLBO | 127 | 84.3 | 51.5 | 5.6 | 21.9 | 30.4 | 40.8 | 13.5 |
|  | CZP | 246 | 83.7 | 52.2 | 6.1 | 20.5 | 30.1 | 43.7 | 14.2 |
|  | CZP | 246 | 78.0 | 51.9 | 6.5 | 21.0 | 30.0 | 39.1 | 13.1 |
| REALISTIC | CZP | 851 | 77.6 | 55.4 | 8.6 | 11.8 | 14.7 | 37.0 (median) | 9.0 (median) |
|  | PLBO | 212 | 79.7 | 53.9 | 8.9 | 11.1 | 14.7 | 40.0 (median) | 10.0 (median) |
| ROSE | PLBO | 205 | 83.9 | 55.8 | 8.5 | 19.9 | 30.4 | 47.3 | 171.4 nmol/l |
|  | TCZ | 409 | 79.5 | 55.2 | 8.6 | 19.7 | 29.7 | 46.0 | 174.3 nmol/l |
| SARIL-RA-MOBILITY PART A | SAR | 52 | 80.8 | 48.7 | 6.0 | 16.6 | 25.5 | NR | 19.0 |
|  | SAR | 51 | 82.4 | 51.2 | 7.7 | 17.6 | 26.9 | NR | 17.6 |
|  | PLBO | 52 | 73.1 | 55.2 | 8.1 | 17.5 | 27.1 | NR | 21.8 |
| SARIL-RA-MOBILITY PART B | SAR | 399 | 85.0 | 50.8 | 8.6 | 16.8 | 26.5 | NR | 2.2 mg/dl |
|  | PLBO | 398 | 81.0 | 50.9 | 9.1 | 16.7 | 26.8 | NR | 2.0 mg/dl |
|  | SAR | 400 | 80.0 | 50.1 | 9.5 | 16.6 | 27.2 | NR | 2.4 mg/dl |
| STAR | ADA | 318 | 79.6 | 55 | 9.3 | 20.9 | 27.3 | NR | 1.5 mg/dl |
|  | PLBO | 318 | 79.2 | 55.8 | 11.5 | 21.3 | 27.6 | NR | 1.5 mg/dl |
| START | IFX | 361 | 77.8 | 52 (median) | 6.3 (median) | 15.00 (median) | 22.00 (median) | NR | 1.6 mg/dl (median) |
|  | IFX | 360 | 80.0 | 53 (median) | 7.8 (median) | 15.00 (median) | 22.00 (median) | NR | 1.6 mg/dl (median) |
|  | PLBO | 363 | 83.2 | 52 (median) | 8.4 (median) | 15.00 (median) | 22.00 (median) | NR | 1.2 mg/dl (median) |
| SUMMACTA | TCZ (SC) | 558 | 82.6 | 52.4 | 8.7 | 15.1 | 27.5 | NR | 2.1 mg/dl |
|  | TCZ (IV) | 537 | 82.7 | 52.5 | 8.7 | 16.8 | 28.8 | NR | 2.2 mg/dl |
| TEMPO | ETN +PLBO | 223 | 77.0 | 53.2 | 6.3 | 23.0 | 35.0 | NR | 324.0 |
|  | PLBO + MTX | 228 | 79.0 | 53 | 6.8 | 22.6 | 33.1 | NR | 225.0 |
|  | ETN + MTX | 231 | 74.0 | 52.5 | 6.8 | 22.1 | 34.2 | NR | 299.0 |
| TOWARD | PLBO | 413 | 84.0 | 54 | 9.8 | 18.7 | 29.1 | 49.2 | 2.6 mg/dl |
|  | TCZ | 803 | 81.0 | 53 | 9.8 | 19.7 | 30.1 | 48.2 | 2.6 mg/dl |

Supplementary Figure 1. ACR20 response at 24 weeks (monotherapy) - Odds ratios and 95% CIs for TOF 5 mg and TOF 10 mg versus other treatments, as obtained with random effects NMA
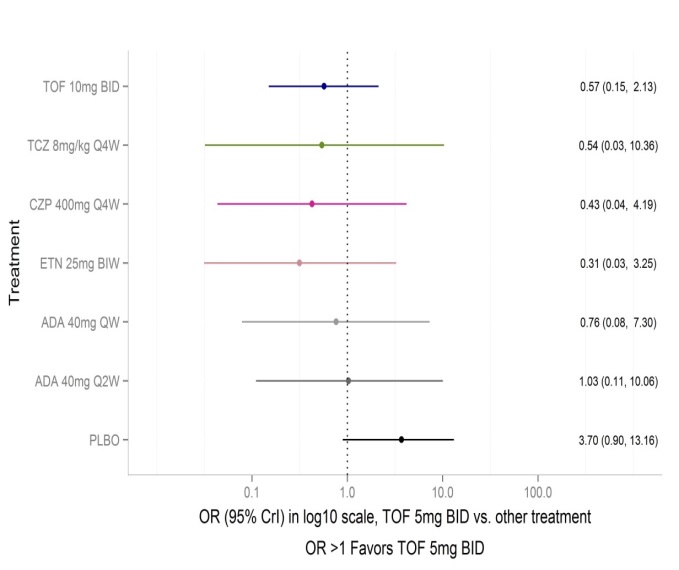

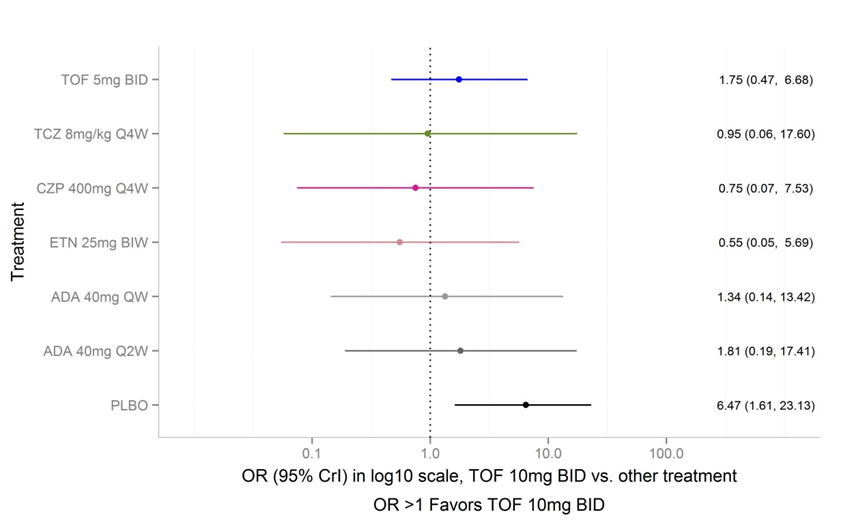


Supplementary Figure 2. ACR50 response at 24 weeks (monotherapy) – Odds ratios and 95% CIs for TOF 5 mg and TOF 10 mg versus other treatments, as obtained with random effects NMA
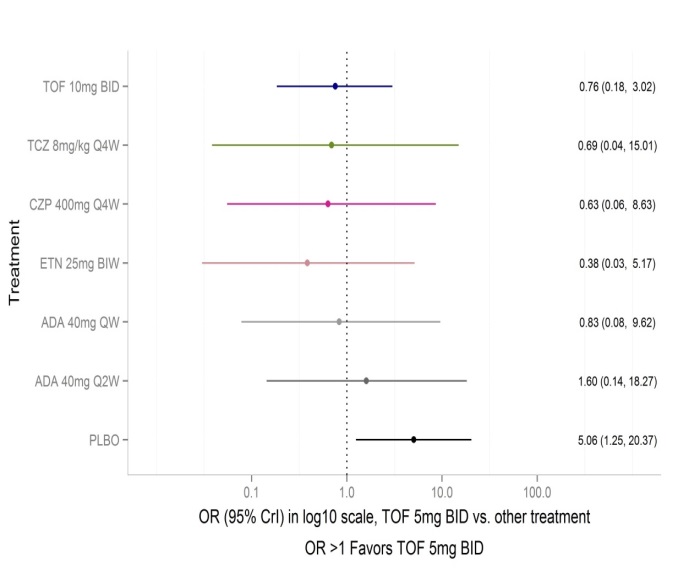

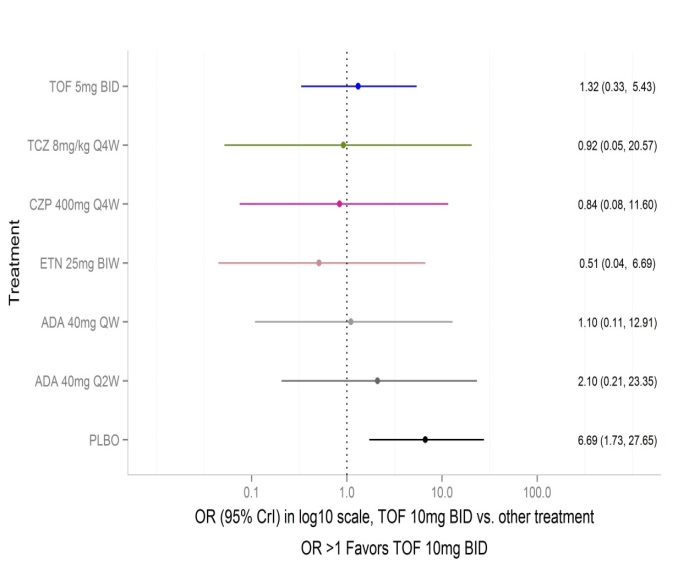


Supplementary Figure 3. ACR70 response at 24 weeks (monotherapy) – Odds ratios and 95% CIs for TOF 5 mg and TOF 10 mg versus other treatments, as obtained with random effects NMA


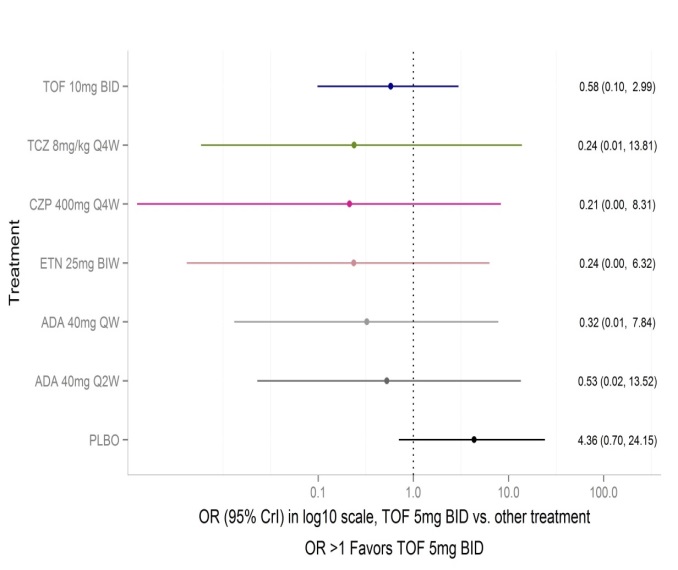

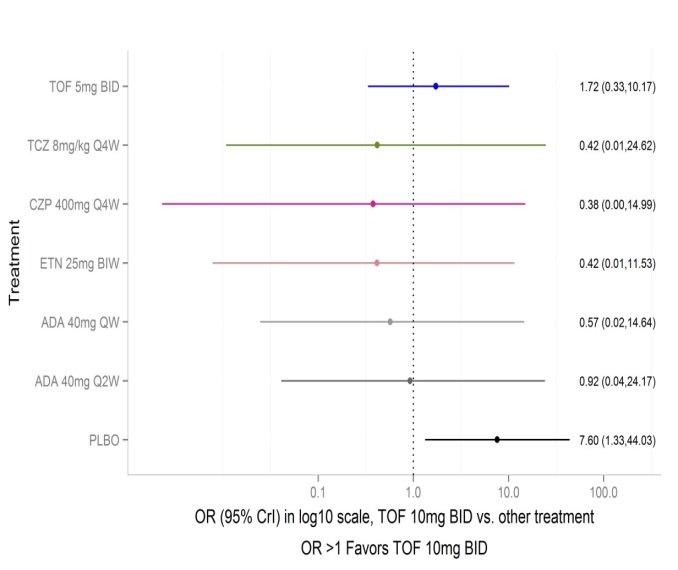


Supplementary Figure 4. Withdrawals due to adverse events (Monotherapy) – Odds ratios and 95% CIs for TOF 5 mg and TOF 10 mg versus other treatments, as obtained with random effects NMA
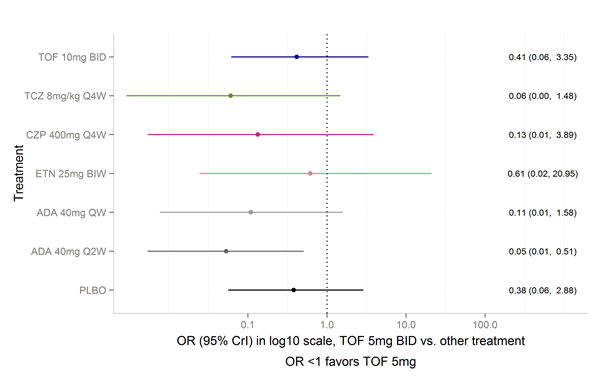

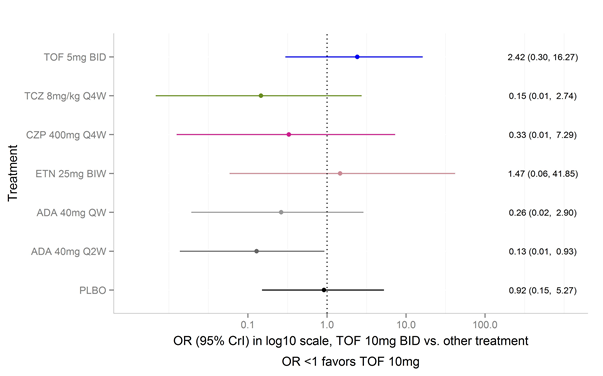


Supplementary Figure 5. ACR20 response at 24 weeks (combination therapy) - Odds ratios and 95% Cls for TOF 5 mg + DMARDs and TOF 10 mg + DMARDs versus other treatments, as obtained with random effects NMA


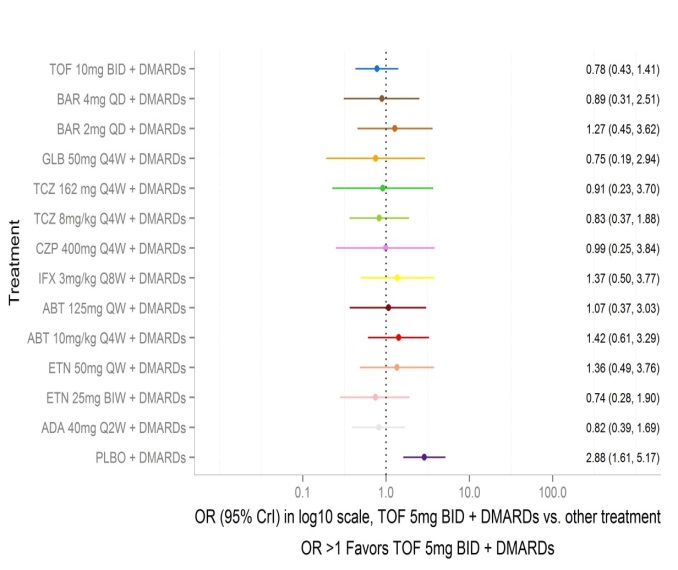

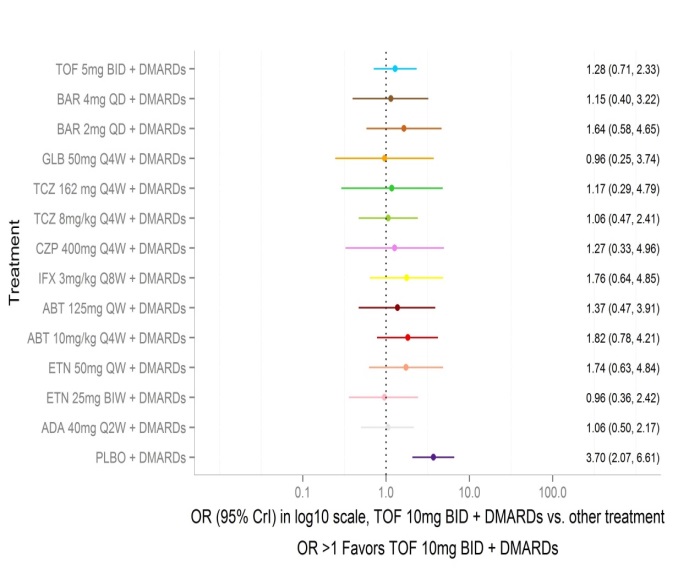


Supplementary Figure 6. ACR50 response at 24 weeks (combination therapy) - Odds ratios and 95% Cls for TOF 5 mg + DMARDs and TOF 10 mg + DMARDs versus other treatments, as obtained with random effects NMA


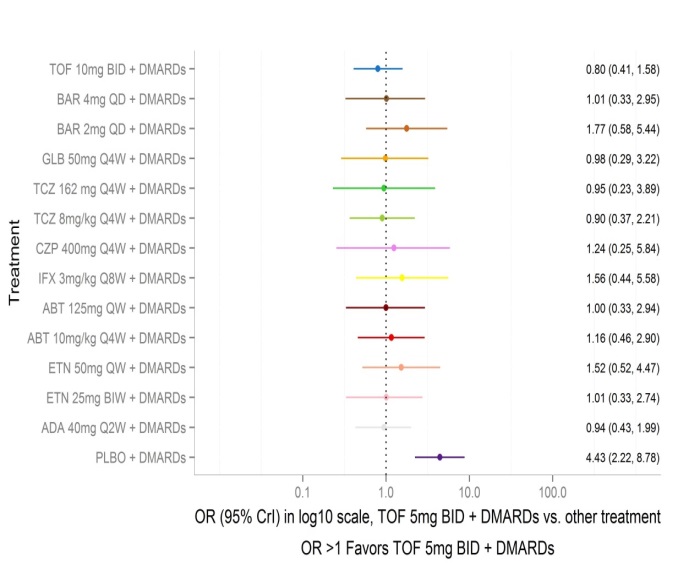

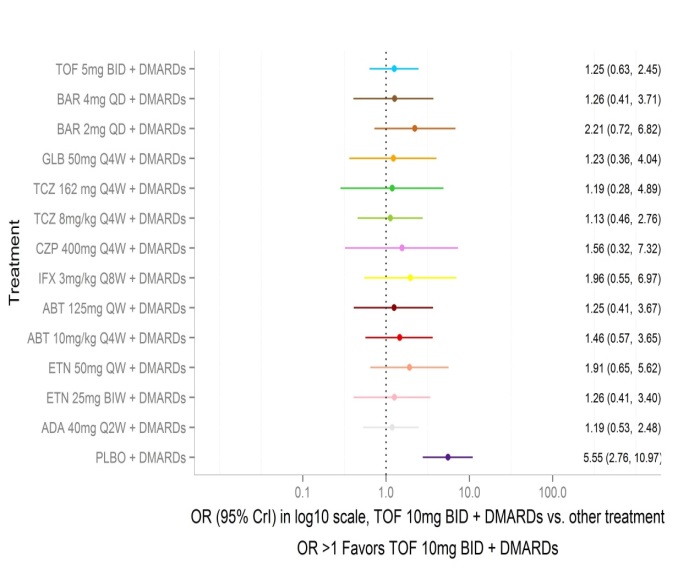


Supplementary Figure 7. ACR70 response at 24 weeks (combination therapy) - Odds ratios and 95% Cls for TOF 5 mg + DMARDs and TOF 10 mg + DMARDs versus other treatments, as obtained with random effects NMA


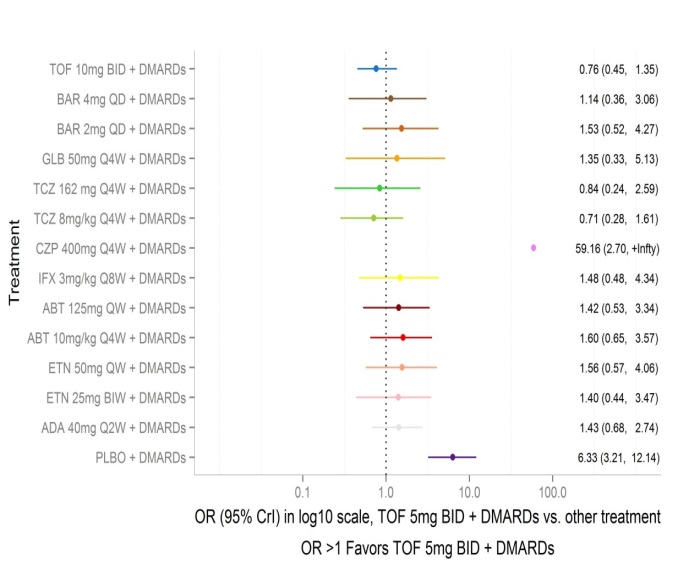

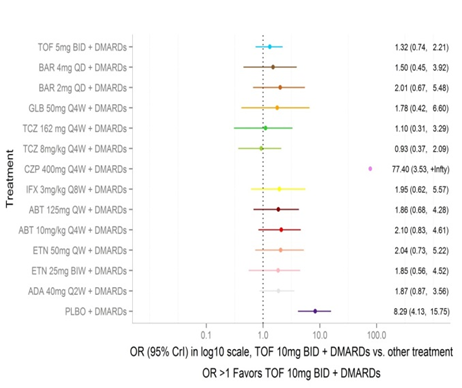


Supplementary Figure 8. HAQ-DI at 24 weeks (Combination therapies) Modelled change from baseline and 95% CIs for all treatments, as obtained with random effects NMA

**
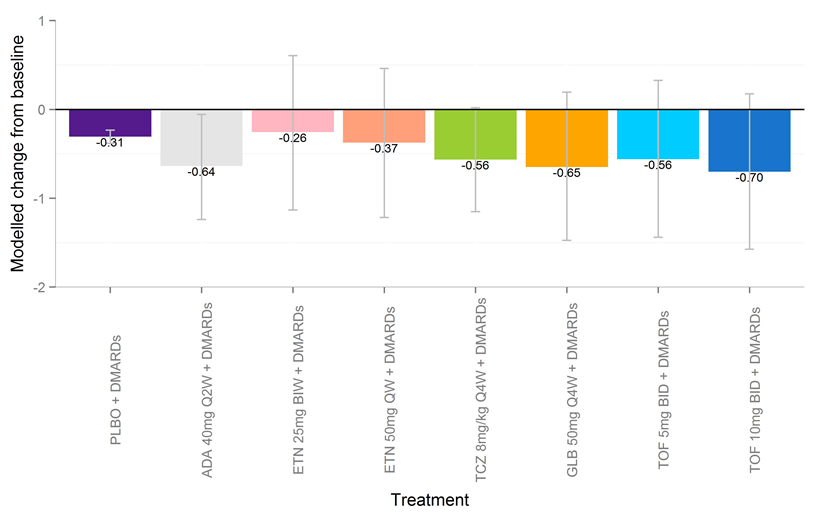
**

Supplementary Figure 9. Withdrawals due to adverse events (Combination therapy) - Odds ratios and 95% Cls for TOF 5 mg + DMARDs and TOF 10 mg + DMARDs versus other treatments, as obtained with random effects NMA


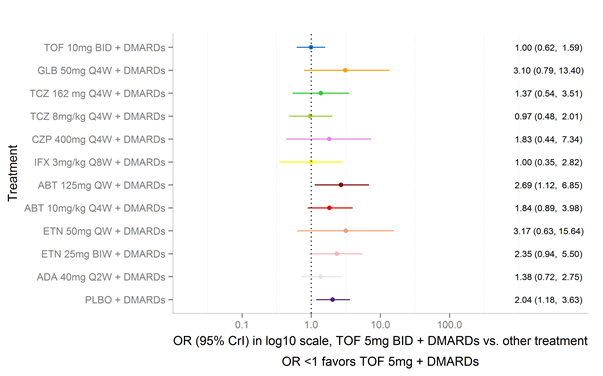

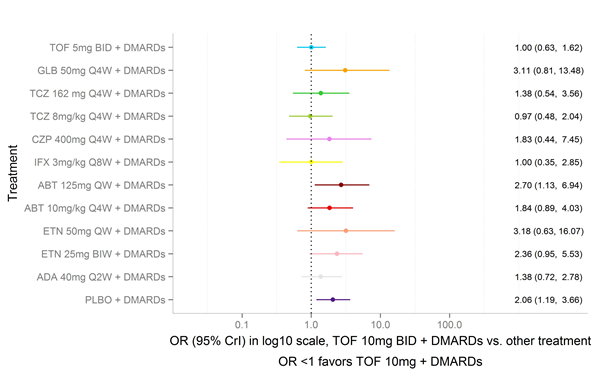


Supplementary Figure 10. ACR20 response at 24 weeks (MTX combination therapy) – Odds ratios and 95% CIs for TOF 5 mg + MTX and TOF 10 mg + MTX versus other treatments, as obtained with random effects NMA


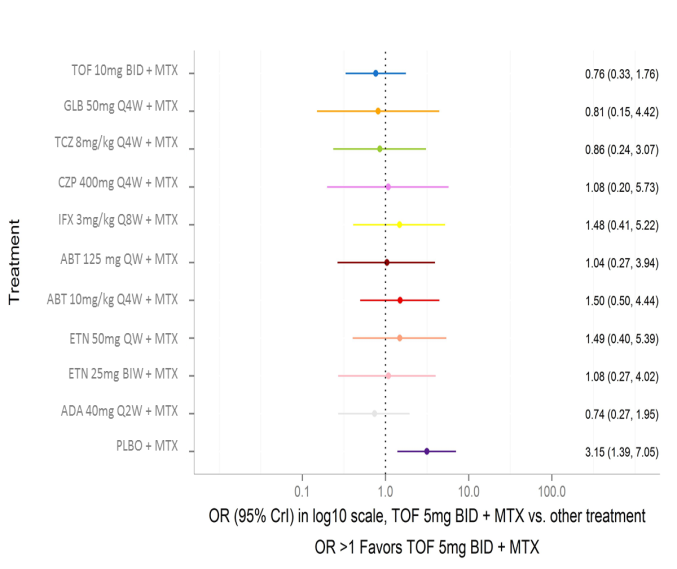

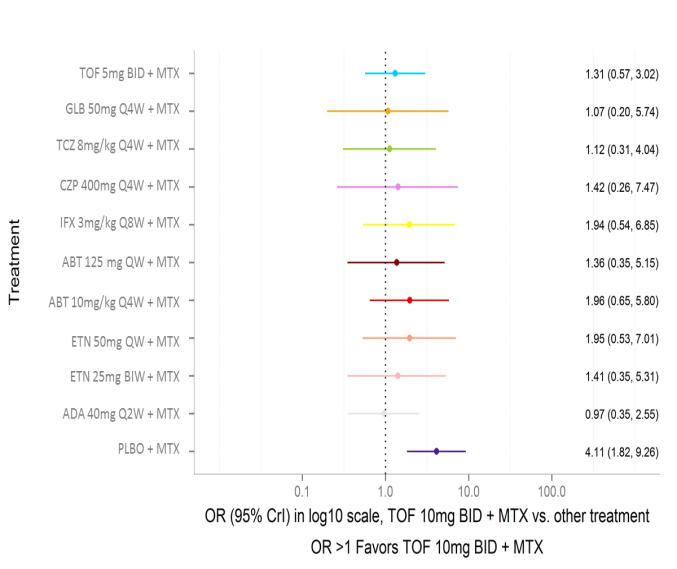


Supplementary Figure 11. ACR50 response at 24 weeks (MTX combination therapy) – Odds ratios and 95% CIs for TOF 5 mg + MTX and TOF 10 mg + MTX versus other treatments, as obtained with random effects NMA


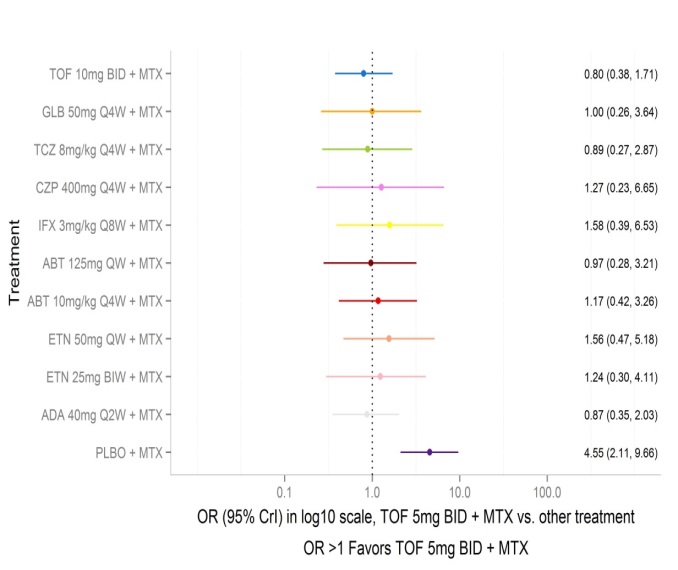

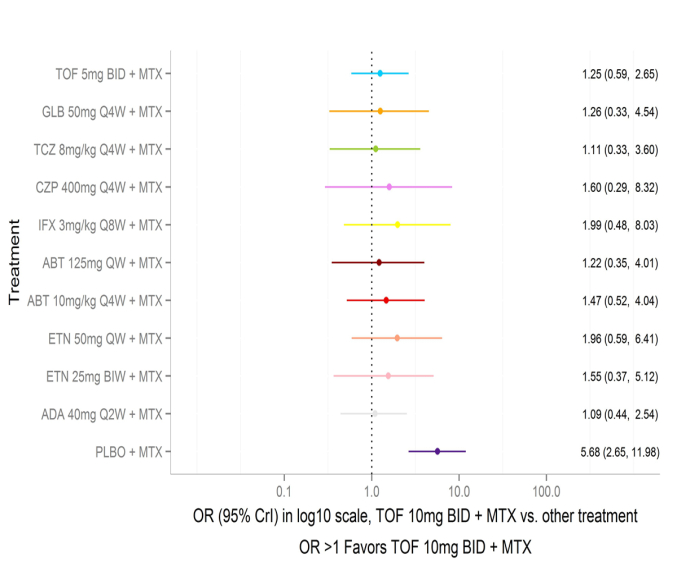


Supplementary Figure 12. ACR70 response at 24 weeks (MTX combination therapy) – Odds ratios and 95% CIs for TOF 5 mg + MTX and TOF 10 mg + MTX versus other treatments, as obtained with random effects NMA


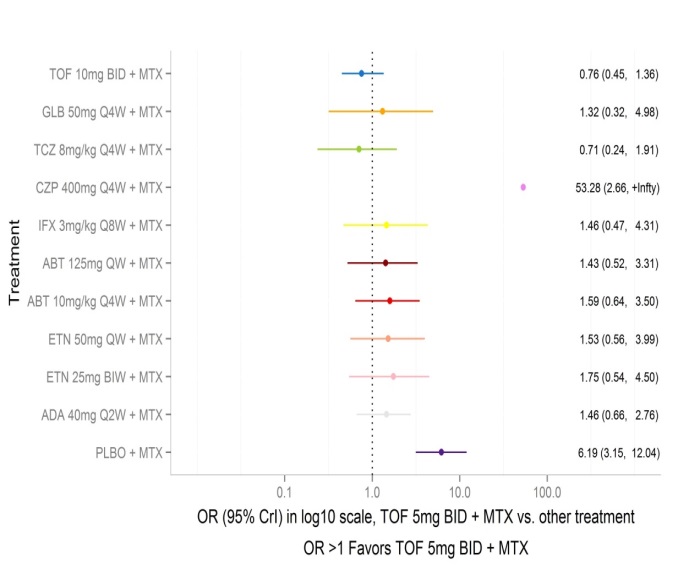

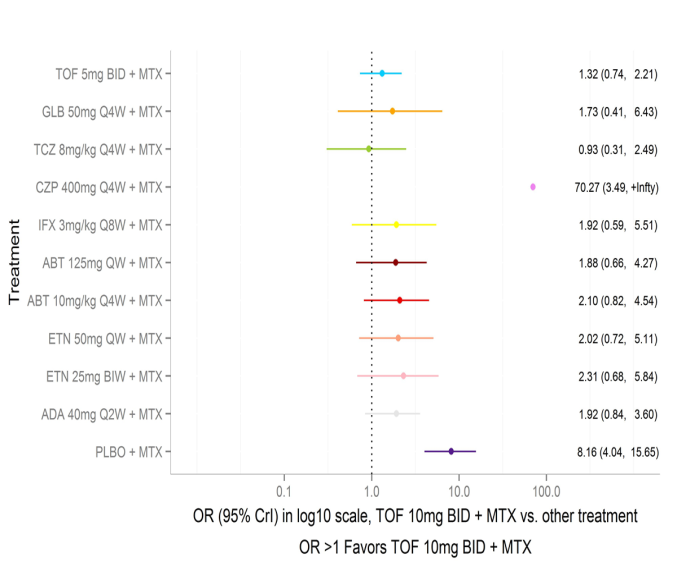


Supplementary Figure 13. HAQ-DI at 24 weeks (MTX combination therapies) - Modelled change from baseline and 95% CIs for all treatments, as obtained with random effects NMA


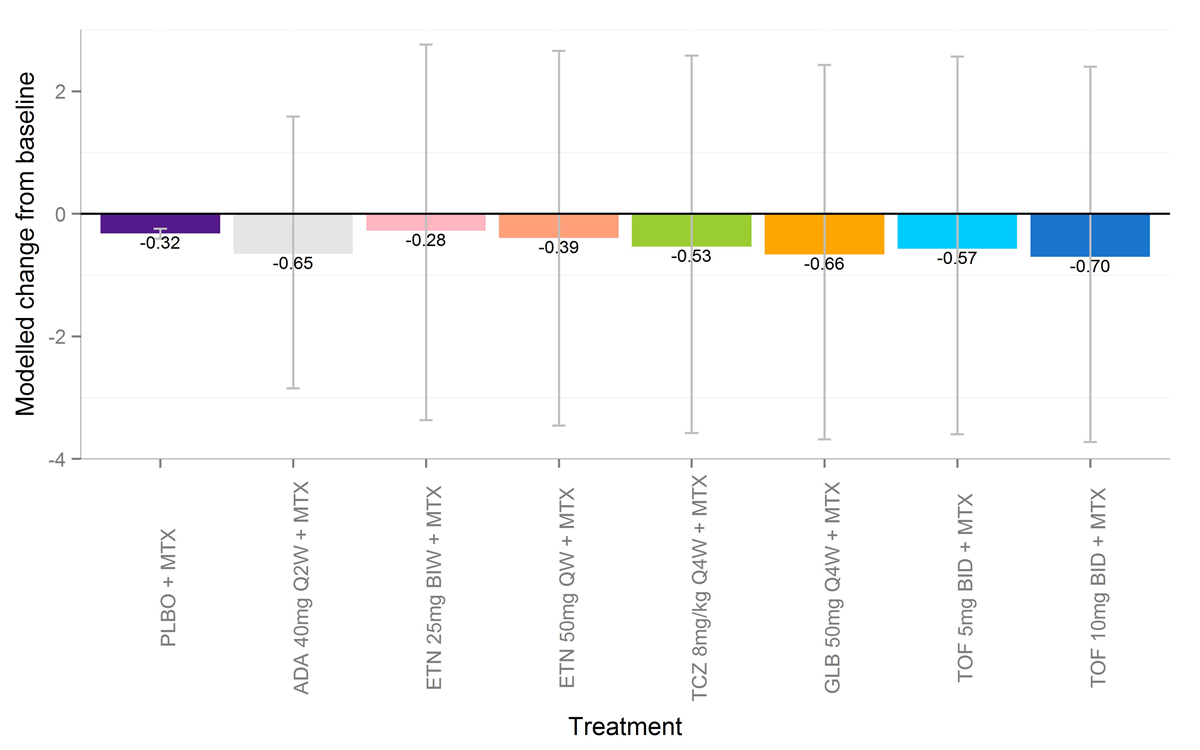


Supplementary Figure 14. Withdrawals due to adverse events (MTX combination therapy) – Odds ratios and 95% CIs for TOF 5 mg + MTX and TOF 10 mg + MTX versus other treatments, as obtained with random effects NMA


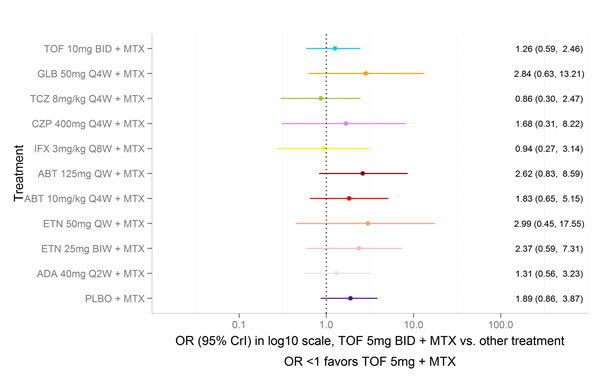

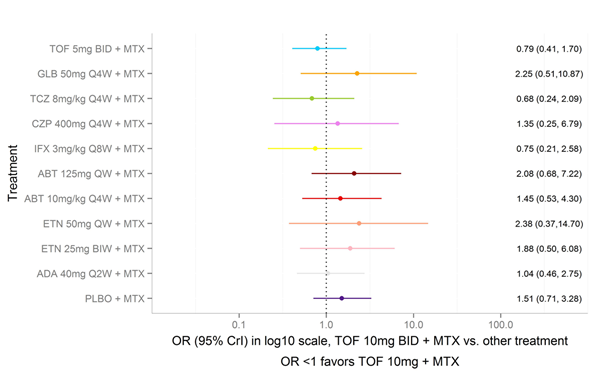

Supplement: Supplementary file 1 — Supplementary Table 1 provides an overview of the patient characteristics across all the identified RCTs, Supplementary Figure 1. ACR20 response at 24 weeks (monotherapy) - Odds ratios and 95% CIs for TOF 5 mg and TOF 10 mg versus other treatments, as obtained with random effects NMA, Supplementary Figure 2. ACR50 response at 24 weeks (monotherapy) – Odds ratios and 95% CIs for TOF 5 mg and TOF 10 mg versus other treatments, as obtained with random effects NMA, Supplementary Figure 3. ACR70 response at 24 weeks (monotherapy) – Odds ratios and 95% CIs for TOF 5 mg and TOF 10 mg versus other treatments, as obtained with random effects NMA, Supplementary Figure 4. Withdrawals due to adverse events (Monotherapy) – Odds ratios and 95% CIs for TOF 5 mg and TOF 10 mg versus other treatments, as obtained with random effects NMA, Supplementary Figure 5. ACR20 response at 24 weeks (combination therapy) - Odds ratios and 95% Cls for TOF 5 mg + DMARDs and TOF 10 mg + DMARDs versus other treatments, as obtained with random effects NMA, Supplementary Figure 6. ACR50 response at 24 weeks (combination therapy) - Odds ratios and 95% Cls for TOF 5 mg + DMARDs and TOF 10 mg + DMARDs versus other treatments, as obtained with random effects NMA, Supplementary Figure 7. ACR70 response at 24 weeks (combination therapy) - Odds ratios and 95% Cls for TOF 5 mg + DMARDs and TOF 10 mg + DMARDs versus other treatments, as obtained with random effects NMA, Supplementary Figure 8. HAQ-DI at 24 weeks (Combination therapies) Modelled change from baseline and 95% CIs for all treatments, as obtained with random effects NMA, Supplementary Figure 9. Withdrawals due to adverse events (Combination therapy) - Odds ratios and 95% Cls for TOF 5 mg + DMARDs and TOF 10 mg + DMARDs versus other treatments, as obtained with random effects NMA, Supplementary Figure 10. ACR20 response at 24 weeks (MTX combination therapy) – Odds ratios and 95% CIs for TOF 5 mg + MTX and TOF 10 mg + MTX versus other treatments, as [file 8417249.f1.docx]
